# Rewiring Photosynthesis by Water‐Soluble Fullerene Derivatives for Solar‐Powered Electricity Generation

**DOI:** 10.1002/advs.202310245

**Published:** 2024-04-22

**Authors:** Huawei Zhu, Franco M. Cabrerizo, Jing Li, Tao He, Yin Li

**Affiliations:** ^1^ CAS Key Laboratory of Microbial Physiological and Metabolic Engineering State Key Laboratory of Microbial Resources Institute of Microbiology Chinese Academy of Sciences Beijing 100101 China; ^2^ Instituto Tecnológico de Chascomús (CONICET‐UNSAM) Av. Intendente Marino Km 8.2, CC 164 (B7130IWA) Chascomús CP7130 Argentina; ^3^ Key Laboratory of Photochemical Conversion and Optoelectronic Materials Technical Institute of Physics and Chemistry Chinese Academy of Sciences Beijing 100190 China; ^4^ CAS Key Laboratory of Nanosystem and Hierarchical Fabrication National Center for Nanoscience and Technology Beijing 100190 China

**Keywords:** biophotovoltaics, extracellular electron extraction, photocurrent, photosynthesis, water‐soluble fullerenes

## Abstract

Natural photosynthesis holds great potential to generate clean electricity from solar energy. In order to utilize this process for power generation, it is necessary to rewire photosynthetic electron transport chains (PETCs) of living photosynthetic organisms to redirect more electron flux toward an extracellular electrode. In this study, a semi‐artificial rewiring strategy, which use a water‐soluble fullerene derivative to capture electrons from PETCs and donate them for electrical current generation, is proposed. A positively charged fullerene derivative, functionalized with *N,N*‐dimethyl pyrrolidinium iodide, is found to be efficiently taken up by the cyanobacterium *Synechocystis* sp. PCC 6803. The distribution of this fullerene derivative near the thylakoid membrane, as well as site‐specific inhibitor assays and transient absorption spectroscopy, suggest that it can directly interact with the redox centers in the PETCs, particularly the acceptor side of photosystem I (PSI). The internalized fullerene derivatives facilitate the extraction of photosynthetic electrons and significantly enhance the photocurrent density of *Synechocystis* by approximately tenfold. This work opens up new possibility for the application of fullerenes as an excellent 3D electron carrier in living biophotovoltaics.

## Graphical abstract

Researchers use a positively charged water‐soluble fullerene derivative to rewire the natural photosynthetic electron transfer process in cyanobacteria. By integrating this novel derivative into the photosynthetic electron transport chains, it captures electrons from photosystems, amplifies the electron flow toward extracellular environments, and dramatically boosts the solar‐powered electrical current generation by one order of magnitude.

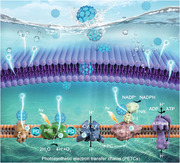

## 1 Introduction

Photosynthesis uses solar energy to convert carbon dioxide into organic matter, which drives most biochemical processes on earth.^[^ ^1^ ^]^ Photosynthetic organisms have developed versatile photosynthetic electron transport chains (PETCs) to perform this crucial energy conversion process. In the PETCs of oxygenic photosynthetic organisms, the oxygen‐evolving complex (OEC) in photosystem II (PSII) catalyzes the water oxidation reaction, generating high‐energy electrons at high quantum efficiency.^[^ ^2^ ^]^ These high‐energy electrons serve as a valuable energy reservoir that can be redirected to various biological and artificial pathways, enabling biotechnological applications such as solar‐powered electricity generation and chemical synthesis.^[^ ^2^ ^]^ Regarding electricity generation, PETCs are generally connected into an external electrode in a bioelectrochemical system.^[^ ^3^ ^]^ Photosynthetic electrons are redirected from PETCs to extracellular environments and flow from the anode to the cathode, thus generating an electrical current.^[^ ^4^ ^]^

Redirecting the native electron transfer pathways in PETCs and transporting electrons out of the photosynthetic cells pose two challenges in solar‐powered electricity generation. The former competes with crucial cellular processes, such as carbon fixation and respiration, for electrons. The latter is trapped by the electron transfer barriers like cell membrane and cell wall that enclose the PETCs.^[^ ^4^ ^]^ To address these issues, various biological rewiring strategies have been proposed in recent years. For instance, removing electron sinks such as flavodiiron or terminal oxidase enzymes increased the power output up to 2–4 times by redirecting intracellular electron flow.^[^ ^5^, ^6^ ^]^ In addition, heterologous expression of heme‐containing cytochromes such as OmcS and MtrA in cyanobacteria exhibited higher photocurrent generation.^[^ ^7^, ^8^ ^]^ In previous studies, we designed and constructed two photosynthetic‐exoelectrogenic microbial consortia to bypass the weak exoelectrogenesis of cyanobacteria, resulting in improved power output and extended system lifespan.^[^ ^9^, ^10^ ^]^ Nonetheless, the significant enhancement of solar‐powered electricity generation remains limited by the electrical conductivity constraints of biological components.

Carbon nanomaterials, such as fullerenes, carbon nanotubes, and graphene have attracted considerable interest from both the scientific and industrial communities due to their low cost, diverse structures, and unique properties, such as excellent electrical conductivity.^[^ ^11^ ^]^ These characteristics also make them suitable for use in the bioelectrochemical systems, where carbon nanomaterials can aid electron transfer across different locations.^[^ ^12^ ^]^ In most studies, carbon nanomaterials have been primarily used to modify the electrode surface for strengthening the electron transfer at the cell‐electrode interface.^[^ ^4^, ^13^ ^]^ However, achieving direct rewiring of intracellular electrons with carbon nanomaterials remains challenging unless cellular uptake and specific distribution are effectively addressed. A recent study found that lysozyme‐coated single‐walled carbon nanotubes (LSZ‐SWCNT) were capable of penetrating the cell walls of photosynthetic cells and showed preferential heterogeneous distribution in the peripheral regions.^[^ ^14^ ^]^ The LSZ‐SWCNT‐treated cells achieved an approximately 15‐fold enhancement of the photocurrent compared with that of untreated cells, showing the great potential of photosynthetic‐carbon nanomaterial hybrids for extracting photosynthetic electrons.

The aim of this work was to directly rewire the redox centers of PETCs using a 0D carbon nanomaterial (fullerenes) for solar‐powered electricity generation. Fullerenes have spherical and hollow cage structures with a diameter of ≈1 nm,^[^ ^11^ ^]^ making them more likely to be taken up by photosynthetic cells compared to other 1D or 2D carbon nanomaterials. Moreover, fullerenes have a high electron affinity and can undergo reversible reduction with up to six electrons.^[^ ^15^ ^]^ One well‐known fullerene derivative, phenyl‐C_61_‐butyric acid methyl ester (PCBM), has been widely used in the field of organic photovoltaics (OPV) as an acceptor in internal donor–acceptor heterojunctions.^[^ ^16^ ^]^ Considering their excellent electron exchange capability, fullerenes and derivatives are desirable components for rewiring PETCs in photosynthetic cells. We found that a positively charged water‐soluble fullerene derivative could enter the cyanobacterium *Synechocystis* sp. PCC 6803 (hereafter referred to as *Synechocystis*) and exhibited a preferred distribution near the thylakoid membrane. This resulted in a significant enhancement in photocurrent generation. To the best of our knowledge, this is the first report that uses fullerene in the field of photosynthetic energy conversion of living organisms.

## 2 Results and Discussion

### 2.1 Characterization of Water‐Soluble Fullerene Derivative

**Figure 1:**
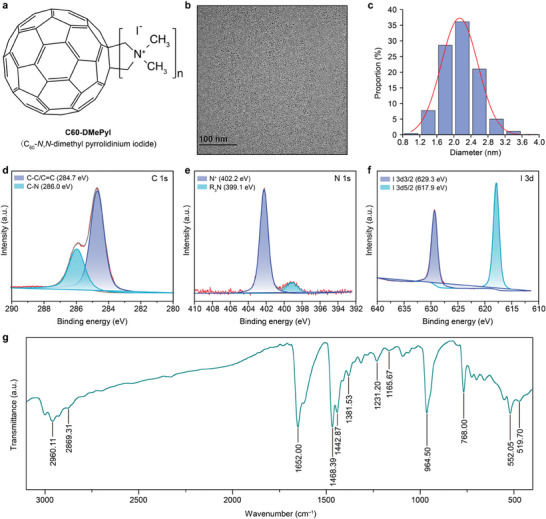
Morphology and composition of C60‐DMePyI. a) Chemical structure of C60‐DMePyI. b) TEM image of C60‐DMePyI nanoparticles. c) The size distribution of C60‐DMePyI nanoparticles. d–f) XPS spectra of C60‐DMePyI. g) FTIR spectrum of C60‐DMePyI.

The inherent structure of pristine fullerenes is highly hydrophobic, whereas water‐solubility is generally a requirement for fullerenes when used in biological systems, including photosynthetic organisms. Functionalizing the surface of fullerene by introducing hydrophilic groups has led to the development of various water‐soluble fullerene derivatives for biomedical applications.^[^ ^17^ ^]^ In this study, we use a water‐soluble fullerene derivative of C_60_‐*N,N*‐dimethyl pyrrolidinium iodide (C60‐DMePyI) as the rewiring material for electricity generation of *Synechocystis* (**Figure** **1a**). The positive charge of C60‐DMePyI strengthens its interaction with the negatively charged cell membrane, facilitating cellular uptake. Moreover, the presence of the *N,N*‐dimethyl functional group increases the tendency of C60‐DMePyI to donate electrons, making it suitable as an electron carrier to transfer electrons from PETCs to the electrodes. Transmission electron microscope (TEM) images revealed that C60‐DMePyI has an average diameter of 2.16 ± 0.41 nm (Figure 1b,c). The chemical composition of C60‐DMePyI was confirmed by X‐ray photoelectron spectroscopy (XPS) and Fourier transform infrared (FTIR) spectroscopy. The C 1s spectrum of C60‐DMePyI showed binding energies at 284.7 and 286.0 eV, corresponding to the bonds of C–C/C═C and C–N, respectively (Figure 1d). The N 1s spectrum showed a binding energy peak at 402.2 eV, indicating the presence of quaternary nitrogen in the pyrrolidine ring (Figure 1e). A second peak with low intensity at a binding energy of 399.1 eV suggested a demethylated pyrrolidine nitrogen, resulting from the degeneration of quaternary nitrogen, counterbalanced by iodide (Figure 1e). The XPS spectrum for I 3d showed double binding energies at 629.3 and 617.9 eV, corresponding to the spin‐orbit splitting of I 3d3/2 and I 3d5/2, respectively (Figure 1f). In contrast, the XPS spectrum of non‐functionalized C_60_ only showed a single peak at a binding energy of 284.0 eV, corresponding to the C–C/C = C bonds (Figure S1a, Supporting Information).

FTIR analysis consistently showed characteristic peaks in the spectrum (Figure 1g). These peaks corresponded to various vibrational modes: the C–H stretching vibration at 2960.11 and 2869.31 cm^−1^, the C═C stretching vibration at 1652.00 cm^−1^, the C–H bending vibration at 1468.39 and 1381.53 cm^−1^, the C–N stretching vibration at 1231.20 cm^−1^, the four typical infrared active vibrational modes T1u of C_60_ scaffold at 1442.87, 1165.67, 552.05, and 519.70 cm^−1^,^[^ ^18^ ^]^ and the CH_3_ rocking vibration of N^+^(CH_3_)_2_ group and CH_2_ rocking mode of pyrrolidine ring at 964.50 and 768.00 cm^−1^, respectively.^[^ ^19^ ^]^ The FTIR analysis of unfunctionalized C_60_ confirmed its characteristic peak at 1630.65 cm^−1^ corresponding to C═C stretching vibration, and those at 1428.80, 1182.15, 574.31, and 526.27 cm^−1^ corresponding to the four typical infrared active vibrational modes of C_60_ scaffold (Figure S1b, Supporting Information). These results confirm that the fullerene derivative C60‐DMePyI used in this study has the correct functionality with *N,N*‐dimethyl functional groups and is counterbalanced by the iodide ion. The introduction of this functional group introduces positive charges on the surface of C60‐DMePyI, as confirmed by the zeta potential measurement (+41.2 ± 1.7 mV).

### 2.2 Cellular Uptake of C60‐DMePyI by *Synechocystis*

**Figure 2:**
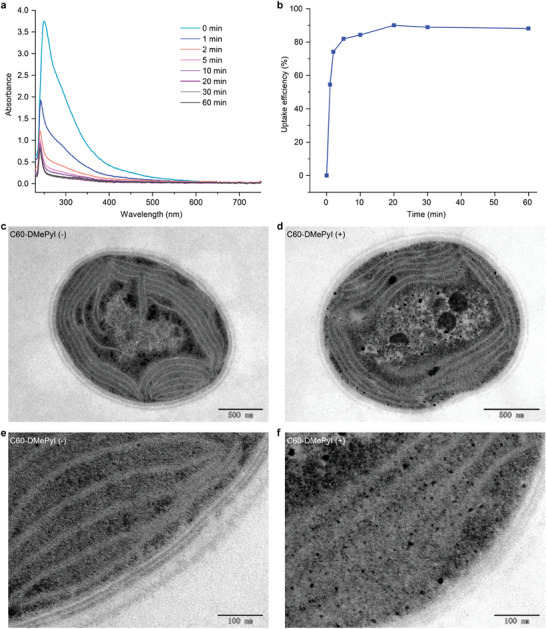
Cellular uptake of C60‐DMePyI by *Synechocystis*. a) The absorption spectra of extracellular residual C60‐DMePyI after incubation with *Synechocystis* cells for different times. b) The uptake efficiency (%), derived from the integral areas of the absorption spectra. c–f) TEM images of cross‐sectional slices of pristine *Synechocystis* cells (c and e) and *Synechocystis* cells treated with C60‐DMePyI (d and f). The large black clusters were assigned to C60‐DMePyI aggregates.

The aqueous solution of C60‐DMePyI exhibited an absorption in the ultraviolet region (**Figure** **2a**), making it suitable for quantifying the cellular uptake efficiency. The fresh cells of *Synechocystis* were resuspended in C60‐DMePyI solution at a concentration of 0.1 mg mL^−1^ and subjected to different incubation times. As shown in Figure 2a,b, the absorption in the supernatants decreased over times, reaching peak uptake efficiency of ≈90% after twenty minutes. This indicates rapid cellular uptake of C60‐DMePyI by *Synechocystis*. The spherical nanostructure and positive surface charge of C60‐DMePyI might play a critical role in driving the electrostatic interaction between C60‐DMePyI and the negatively charged cell envelope, resulting in efficient cellular uptake similar to other fullerene derivatives.^[^ ^20^ ^]^ Transmission electron microscopy (TEM) was conducted to track the cellular distribution of C60‐DMePyI in cross‐section cell slices. The TEM images of pristine *Synechocystis* cells clearly showed the cell periphery, central cytoplasm, and an array of internal thylakoid membranes connected to the plasma membrane at convergence sites (Figure 2c,e). After treatment with C60‐DMePyI, the TEM images showed dense dark spots dispersed inside *Synechocystis* cells, corresponding to C60‐DMePyI nanoparticles or clusters (Figure 2d,f). These nanoparticles mainly distributed around the thylakoid membranes and also within the cytoplasm. Notably, there was no significant distribution in cell envelope, such as periplasmic space and outer membrane. These results suggest efficient cellular uptake and preferential distribution of C60‐DMePyI in *Synechocystis* cells. Attempts were made to further investigate the cellular distribution of C60‐DMePyI by labeling with fluorescein isothiocyanate (FITC), while no fluorescence was observed after FITC was linked to the fullerene cages. This would be attributed to the fluorescence quenching between fluorescent molecules and fullerene cages.^[^ ^21^ ^]^

### 2.3 Enhanced Photocurrent Generation by C60‐DMePyI Rewiring

The effect of C60‐DMePyI on photocurrent generation of cyanobacterium *Synechocystis* was investigated in biophotovoltaic systems. Photocurrent measurements were conducted in a single‐chamber electrochemical device with a three‐electrode configuration by depositing the *Synechocystis* cells onto the indium tin oxide (ITO) glass. Monochromatic red light (λ = 658 nm) was used as the source of irradiation, as it can be absorbed by the antenna proteins of *Synechocystis* but cannot be absorbed by C60‐DMePyI (Figure S2, Supporting Information). Stepped chronoamperometry scans showed the photocurrent response at different applied potentials under chopped light irradiation (Figure S3, Supporting Information). In this photocurrent profile, an anodic photocurrent with an onset potential of 0.5 V (vs Ag/AgCl, the same hereafter) was observed. The highest photocurrent response occurred at an applied potential of 0.8 V for both the *Synechocystis* alone (labeled as Syn6803) and the *Synechocystis* treated with C60‐DMePyI (labeled as Syn6803+C60‐DMePyI). The bare ITO glass electrode did not produce any photocurrent under the same conditions (Figure S3, Supporting Information). Further photocurrent measurement was performed at an applied potential of 0.8 V under the light/dark cycles.

**Figure 3:**
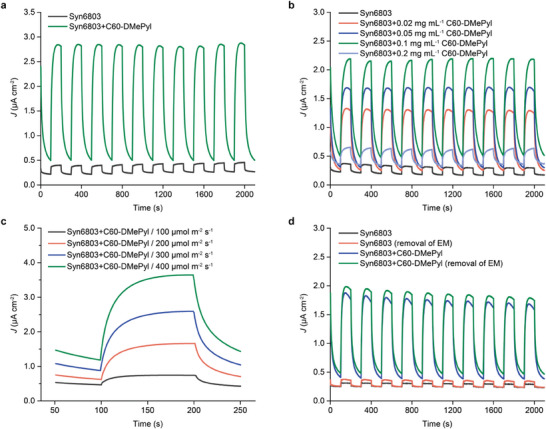
Enhanced photocurrent generation of *Synechocystis* cells by rewiring with C60‐DMePyI. a) Photocurrent generation of *Synechocystis* cells in the absence or presence of C60‐DMePyI. b) Photocurrent generation of *Synechocystis* cells treated with C60‐DMePyI at different concentrations. c) Photocurrent generation of *Synechocystis* cells treated with C60‐DMePyI under different light intensities. d) Photocurrent generation upon removal of extracellular residual C60‐DMePyI. EM indicates extracellular medium. The label of Syn6803 represents *Synechocystis* sp. PCC 6803. The chopped irradiation (100 s light/100 s dark cycles) was used in photocurrent measurement.

As shown in **Figure** **3a**, the photocurrent density generated by the *Synechocystis* cells treated with C60‐DMePyI was ≈2.3 µA cm^−2^, which was about tenfold higher than that of the *Synechocystis* cells alone. The greatest enhancement in photocurrent density was achieved when feeding the *Synechocystis* cells with C60‐DMePyI nanoparticles at an optimized concentration of 0.1 mg mL^−1^ (Figure 3b). Photocurrent density improved as the light intensity increased (Figure 3c; Figure S4, Supporting Information). For the *Synechocystis* alone, the photocurrent density increased twofold when the light intensity was increased from 100 to 400 µmol photons m^−2^ s^−1^ (Figure S4, Supporting Information). In contrast, the photocurrent density generated by the *Synechocystis* cells treated with C60‐DMePyI improved by more than tenfold (Figure 3c). This difference indicates that the intrinsic electron transfer pathways of *Synechocystis* cannot fully export intracellular high‐energy electrons, but this process can be facilitated by C60‐DMePyI rewiring. To eliminate the interference of any extracellular residual C60‐DMePyI, the extracellular medium (EM) was removed by centrifugation after cellular uptake was complete. As expected, the photocurrent densities did not decrease upon removal of the EM (Figure 3d), indicating that any residual C60‐DMePyI in the extracellular environment is minimal or does not contribute to the enhancement of photocurrent. In addition to *Synechocystis*, C60‐DMePyI was also capable of rewiring non‐coccoid cyanobacteria species, such as the rod‐shaped *Synechococcus elongatus* PCC 7942, to enhance photocurrent generation by more than tenfold (Figure S5, Supporting Information). This indicates the rewiring strategy using C60‐DMePyI was generally effective in cyanobacteria, irrespective of cellular morphologies. These results demonstrate that rewiring cyanobacterial cells using internalized C60‐DMePyI nanoparticles led to a significant enhancement in extracellular extraction of photosynthetic electrons.

We further evaluated the long‐term performance of photocurrent generation. Throughout the entire measurement of thirty light/dark cycles (≈2 h), both the *Synechocystis* alone and the *Synechocystis* treated with C60‐DMePyI demonstrated excellent stability in generating photocurrents (Figure S6a, Supporting Information). After allowing the electrochemical devices to sit at room temperature for 12 h, both systems still generated ≈70% of the initial photocurrents and exhibited good stability over thirty‐cycle measurement (Figure S6b, Supporting Information). Furthermore, we investigated the cytotoxicity of C60‐DMePyI at different concentrations. Spot assays showed that exogenous C60‐DMePyI was non‐toxic at concentrations lower than 0.05 mg mL^−1^ (Figure S7a, Supporting Information). However, when the concentration increased to 0.1 mg mL^−1^, it had an adverse effect on *Synechocystis*, indicating cytotoxicity. Nevertheless, the enhancement of photocurrent through C60‐DMePyI rewiring was not a result of inhibited growth or metabolic activities of cyanobacterial cells, as the non‐toxic concentrations of 0.02 and 0.05 mg mL^−1^ also led to photocurrent enhancement (Figure 3b). Moreover, fluorescence staining using propidium iodide (PI) was applied to assess cell membrane integrity. PI is a membrane impermeable DNA intercalator that only penetrates damaged membranes and indicates the dead cells. The control group for dead cells consisted of *Synechocystis* cells treated with 70% isopropanol. For all cells treated with C60‐DMePyI, the PI fluorescence remained low, similar to that of pristine cells (Figure S7b, Supporting Information), suggesting that the integrity of cell membranes was not affected by C60‐DMePyI.

### 2.4 Determining the Rewiring Sites of C60‐DMePyI in PETCs

**Figure 4:**
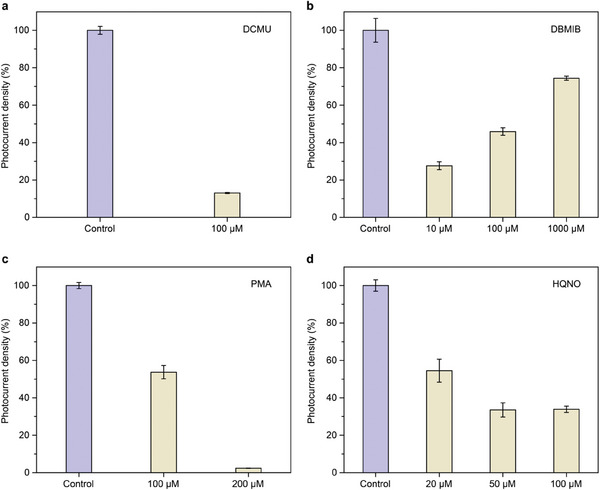
The effects of site‐specific inhibitors on photocurrent generation of *Synechocystis* cells treated with C60‐DMePyI. a) Photocurrent generation under DCMU inhibition. b) Photocurrent generation under DBMIB inhibition. c) Photocurrent generation under PMA inhibition. d) Photocurrent generation under HQNO inhibition. Data are presented as mean values ± SD from *n* =  3 biological replicates.

Based on the preferential distribution of C60‐DMePyI in the thylakoid membrane, we speculate that the enhancement of photocurrent in *Synechocystis* is due to the direct connection between C60‐DMePyI and the components of PETCs. To determine the specific sites where C60‐DMePyI acts in PETCs, a set of electron transfer inhibitors, each specifically targeting different components of PETCs, were used. One of these inhibitors, 3‐(3,4‐dichlorophenyl)−1,1‐dimethyl urea (DCMU), interrupts the electron transfer between Q_A_ and Q_B_ in PSII, thus hindering the linear electron transfer from PSII into the plastoquinone (PQ) pool. The addition of 100 µM DCMU resulted in almost complete elimination of the photocurrents in *Synechocystis* cells, regardless of the presence or absence of C60‐DMePyI (**Figure** **4a**; Figure S8a, Supporting Information). This finding confirms that the photocurrent was originated from the water‐splitting reaction in PSII. The remaining photocurrent might derive from the respiratory electron transfer chains (RETCs) that are linked to the photosynthetic apparatus, as previous studies suggested.^[^ ^22^, ^23^ ^]^ Another inhibitor, 2,5‐dibromo‐3‐methyl‐6‐isopropylbenzoquinone (DBMIB), specifically binds to the quinol oxidation site on the cytochrome *b*_6_ *f* (Cyt *b*_6_ *f*) complex, preventing the electron transfer from the PQ pool into the Cyt *b_6_f* complex.^[^ ^24^ ^]^ We investigated the effect of various concentrations of DBMIB on the photocurrent generation of *Synechocystis*. In the case of *Synechocystis* alone, the photocurrents decreased by 20% at a low concentration of 10 µM compared to the control without DBMIB, but increased by 100–200% at higher concentrations (Figure S8b, Supporting Information). Similar effects were observed in other studies, with one possible explanation being that DBMIB acts as an electron mediator to increase the photocurrent.^[^ ^25^, ^26^ ^]^ Nevertheless, the increase in photocurrent under DBMIB inhibition indicated the electrons were probably redirected from the upper sites of Cyt *b_6_f* complex to the electrode. Unlike *Synechocystis* alone, all treatments with DBMIB, whether at low or high concentrations, resulted in a decrease of photocurrents generated by *Synechocystis* cells treated with C60‐DMePyI (Figure 4b). Among three treatments, the lowest concentration of 10 µM exhibited the highest inhibition, which could be due to its side role as an electron mediator at high concentrations. The significant inhibition with DBMIB indicates the rewiring sites of C60‐DMePyI were located downstream of Cyt *b_6_f* complex.

Phenylmercuric acetate (PMA) is a photosystem I (PSI)‐specific inhibitor that interferes with electron transfer around ferredoxin (Fd), especially from PSI to Fd.^[^ ^27^, ^28^ ^]^ A significant photocurrent increase was observed in PMA‐treated *Synechocystis* (Figure S8c, Supporting Information). This was consistent with the photocurrent increase induced by DBMIB treatment, thus further confirming that the photocurrent of pristine *Synechocystis* originated upstream of PSI. Addition of 200 µM PMA to the *Synechocystis* treated with C60‐DMePyI resulted in nearly complete suppression of photocurrent generation (Figure 4c). This infers that the rewiring sites of C60‐DMePyI were located downstream of PSI acceptor side, most likely being Fd or ferredoxin‐NADP^+^ reductase (FNR). Furthermore, we purified the Fd of *Synechocystis* (Figure S9a, Supporting Information) and performed an in vitro redox reaction under anaerobic conditions. The oxidized Fd had a brown color and exhibited absorption peaks at 330, 420, and 460 nm (Figure S9b, Supporting Information), similar to Fd found in other species such as spinach and *Escherichia coli*.^[^ ^29^, ^30^ ^]^ When Fd was reduced by dithionite, these characteristic peaks disappeared. It was found that the dithionite‐reduced Fd could be reoxidized by C60‐DMePyI and the characteristic peaks reappeared. This result demonstrated the feasibility of C60‐DMePyI to divert electrons from reduced Fd in *Synechocystis* cells. A previous study found that cytochrome *bd*‐type quinol oxidase (Cyd) in RETCs also contributed to the photocurrent generation in cyanobacteria, including the genera of *Nostoc* and *Lyngbya*.^[^ ^23^ ^]^ We further used the inhibitor 2‐heptyl‐4‐hydroxy‐quinoline‐*N*‐oxide (HQNO) to specifically block the electron transfer pathway from PQ pool to Cyd.^[^ ^31^ ^]^ Addition of HQNO at different concentrations significantly decreased photocurrent densities, with maximum inhibition efficiency reaching ≈90% (Figure S8d, Supporting Information). This indicates that the Cyd‐mediated respiration pathway is a primary route for redirecting intracellular electrons to extracellular electrode in pristine *Synechocystis*. However, the photocurrent densities of the *Synechocystis* treated with C60‐DMePyI decreased by 65% under HQNO inhibition (Figure 4d), suggesting that photocurrent generation partially relies on the Cyd‐mediated respiration pathway in the presence of C60‐DMePyI rewiring.

To explore whether C60‐DMePyI interacted directly with photosystems in PETCs, we measured the photochemical efficiencies both in PSII and PSI using pulse‐amplitude modulated (PAM) fluorometry and saturation pulse technology. It was shown that both the effective photochemical quantum yields, Y(II) for PSII and Y(I) for PSI, had a slight decrease in the presence of C60‐DMePyI (Figure S10, Supporting Information). Moreover, there was an slight increase in quantum yield Y(ND) of non‐photochemical energy dissipation due to the donor side limitation of PSI (Figure S10b, Supporting Information). This might be induced by C60‐DMePyI rewiring between PSII and PSI, diverting a portion of photosynthetic electrons before reaching PSI. Furthermore, OJIP fluorescence transient analysis was applied to gain further insights into the behavior of PSII (Figure S11 and Table S1, Supporting Information).^[^ ^32^ ^]^ The decrease in the maximum quantum yield for primary photochemistry (φ_Po_), accompanied by an increase in the quantum yield for energy dissipation (φ_Do_), indicated that a fraction of electrons might be redirected by C60‐DMePyI upstream of the primary electron acceptor Q_A_. However, the efficiency of electron transport beyond Q_A_ (ψ_o_, φ_Eo_) remained unaffected by C60‐DMePyI rewiring. We next measured the steady‐state fluorescence spectra of whole cells of *Synechocystis* under open (*F*_0_) and closed (*F*_M_, in the presence of DCMU) PSII reaction centers. The excitation wavelengths of 435, 475, and 570 nm were used to selectively excite chlorophyll *a* (Chl *a*), chlorophyll *b* (Chl *b*), and phycobilisome (PBS), respectively.^[^ ^33^ ^]^ The fluorescence emission spectra of the *Synechocystis* treated with C60‐DMePyI under *F*_0_ status showed no significant variation when compared to *Synechocystis* alone, while it reduced under *F*_M_ status (Figure S12a,c,e, Supporting Information). This result further demonstrated that C60‐DMePyI might directly connect to PSII and accept electrons upstream of Q_A_, thus reducing the electrons that originally used for energy dissipation to fluorescence. It also explains the previous results that the photocurrent generated by the *Synechocystis* treated with C60‐DMePyI under DCMU inhibition was higher than that of *Synechocystis* alone (Figure 4a; Figure S8a, Supporting Information). In addition, the variable fluorescence (*F*_V_ = *F*_M_ − *F*_0_) exhibited a maximal emission at ≈680 nm regardless of excitation wavelength used (Figure S12b,d,f, Supporting Information), which has been found in other study.^[^ ^34^ ^]^ The maximum quantum yield (*F*_V_/*F*_M_) derived from steady‐state fluorescence spectroscopy also showed a slight decrease (Figure S13, Supporting Information), which was consistent with that using saturation pulse technology. Together, these results suggest the diversion of electrons by C60‐DMePyI from PSII, although the exact rewiring sites remain further exploration.

**Figure 5:**
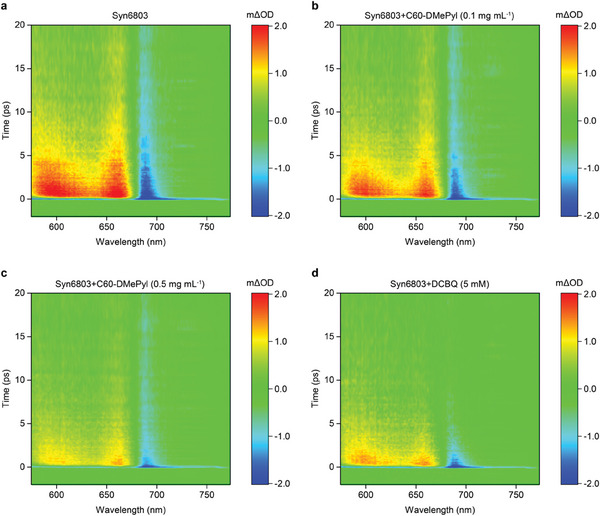
Ultrafast TA spectroscopy map of *Synechocystis* cells between −2 and 20 ps excited at 440 nm in units of differential absorption (m∆OD). a) Pristine *Synechocystis* cells. b) The *Synechocystis* cells treated with 0.1 mg mL^−1^ C60‐DMePyI. c) The *Synechocystis* cells treated with 0.5 mg mL^−1^ C60‐DMePyI. d) The *Synechocystis* cells treated with 5 mm DCBQ. Maps are one sample representative of three biological replicates.

To better understand the electron transfer mechanisms between photosystems and C60‐DMePyI, we performed in vivo ultrafast transient absorption (TA) spectroscopy. When *Synechocystis* cells were photo‐excited with a 25 fs pump pulse centered at 440 nm, a prominent negative spectral feature centered on 690 nm was observed in the TA spectrum of wild‐type *Synechocystis* (**Figure** **5**a). Based on a previous study involving PSII/PSI‐less mutant cells,^[^ ^35^ ^]^ this negative feature was likely associated with to the ground‐state bleach signal of PSI rather than PSII. Furthermore, the positive spectral features below 680 nm were attributed to the photo‐induced absorption of PBS and carotenoids. We found that the internalization of C60‐DMePyI into cyanobacterial cells led to an accelerated decay of the negative feature at 690 nm, including positive features as well (Figure 5b,c). This effect closely resembled the action of 2,6‐dichloro‐1,4‐benzoquinone (DCBQ), which induced a more rapid decay dynamics than C60‐DMePyI (Figure 5d; Figure S14 and Table S2, Supporting Information). Since there was no spectral overlap between the absorption of C60‐DMePyI and the fluorescence of *Synechocystis* (Figures S2 and S12, Supporting Information), it was determined that energy transfer in the form of Förster resonances from the photosynthetic reaction centers to C60‐DMePyI was unlikely. This suggests that the excited state decay acceleration mediated by C60‐DMePyI could be attributed to an electron transfer mechanism. Similar to DCBQ or methyl viologen,^[^ ^35^ ^]^ C60‐DMePyI most likely oxidized the highly delocalized excited state of peripheral chlorophyll pigments protruding from the photosystems, especially PSI.

**Figure 6:**
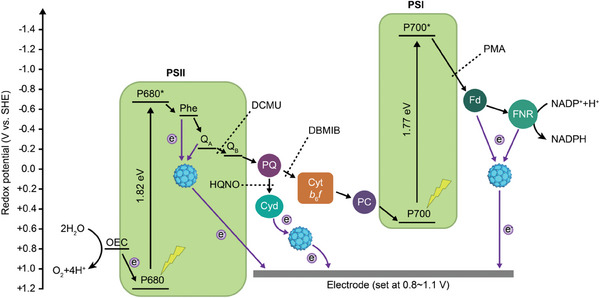
Schematic diagram illustrating the rewiring routes of C60‐DMePyI in PETCs. Photosynthetic electron transfer chains (PETCs) consist of photosystem II (PSII), plastoquinone (PQ), cytochrome *b*_6_ *f* (Cyt *b*_6_ *f*) complex, plastocyanin (PC), photosystem I (PSI), ferredoxin (Fd), and ferredoxin‐NADP^+^ reductase (FNR). The process of photosynthetic electron transfer begins with the water oxidation catalyzed by oxygen evolution center (OEC) in PSII upon photoexcitation. The excited electrons are then transferred along the Z‐scheme electron transfer chain and eventually used for NADPH regeneration. C60‐DMePyI possibly redirected photosynthetic electrons in three sites, including redox centers of PSII upstream of Q_A_, the acceptor side of PSI such as Fd or FNR, and Cyd‐mediated respiration pathway. These routes were indicated by purple arrows. The specific acting sites of different inhibitors were indicated with black dotted lines. The energy level diagram of Z‐scheme electron transfer chain was provided.

Based on aforementioned results, a schematic diagram illustrating the rewiring routes of C60‐DMePyI in PETCs was proposed (**Figure** **6**). The process of photosynthetic electron transfer begins with the water oxidation catalyzed by OEC in PSII upon photoexcitation. The excited electrons are initially accepted by the primary electron acceptor pheophytin (Phe), and then transferred along the Z‐scheme electron transfer chain, which consists of PQ pool, Cyt *b*_6_ *f* complex, plastocyanin (PC), PSI, Fd, and FNR. There are three possible routes to redirect photosynthetic electrons by the internalized C60‐DMePyI. These routes include direct rewiring at the redox centers of PSII upstream of Q_A_, rewiring on the acceptor side of PSI such as Fd or FNR, and rewiring in the Cyd‐mediated respiration pathway. Due to its high electron‐accepting capacity that can be reversibly reduced by up to six electrons, C60‐DMePyI nanoparticles are capable of capturing electrons from these various donors by adjusting their redox potentials. In view of the limited presence of C60‐DMePyI nanoparticles in the cell envelope, it is possible that photosynthetic electrons could be redirected toward intrinsic exoelectrogenesis pathways once they are deviated from the original Z‐scheme electron transfer chain. This hypothesis assumes a direct association between C60‐DMePyI and exoelectrogenic components, although the essential components responsible for exoelectrogenesis have yet to be identified.^[^ ^4^ ^]^

### 2.5 Fullerene Rewiring Depends on Surface Charge and Redox Properties

**Figure 7:**
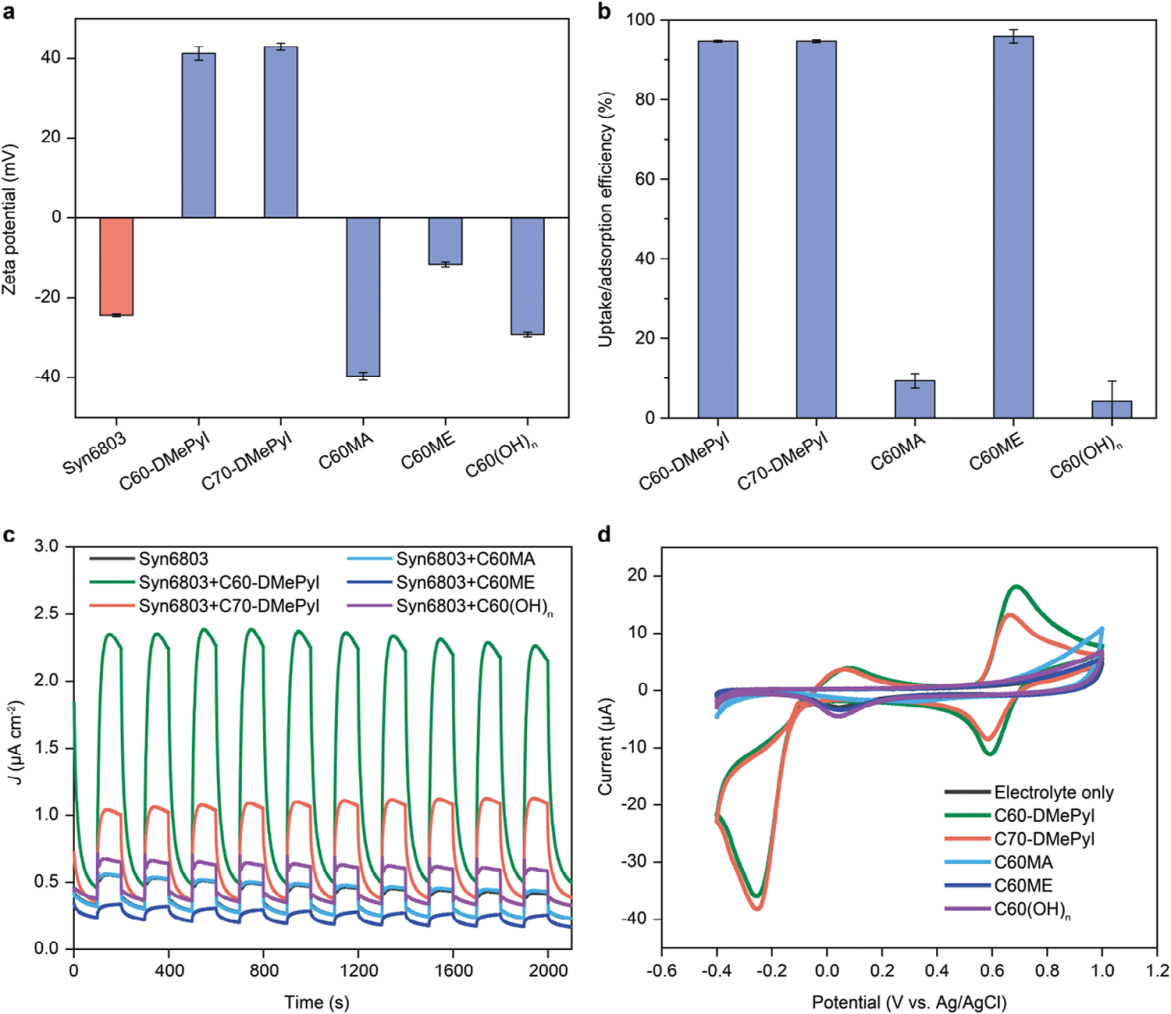
Rewiring photosynthesis with different fullerene derivatives. a) Zeta potentials of *Synechocystis* cells and five fullerene derivatives. b) Cellular uptake/adsorption efficiency of five fullerene derivatives by *Synechocystis* cells. c) Photocurrents generated by the *Synechocystis* cells treated with different fullerene derivatives. d) Cyclic voltammetry (CV) analysis of fullerene derivatives in aqueous solutions. The data are presented as mean values ± SD from independent biological replicates (*n* =  3).

To explore the effect of physicochemical properties of fullerene derivatives on the efficiency of photocurrent enhancement, four additional fullerene derivatives were used for comparison. These includes C_70_‐*N,N*‐dimethyl pyrrolidinium iodide (C70‐DMePyI), C_60_‐malonic acid (C60MA), C_60_‐malonic ester (C60ME) and fullerenol/polyhydroxylated C_60_ (C60(OH)_n_) (Figure S15, Supporting Information). The derivative C70‐DMePyI has same functional group but different cage with C60‐DMePyI. Other three derivatives were functionalized by different charge groups on the surface of C_60_ cage. All of these fullerene derivatives, except for C60ME, exhibited good water‐solubility. Zeta potential measurements showed that both C60‐DMePyI (41.2 ± 1.7 mV) and C70‐DMePyI (42.9 ± 0.9 mV) carried a positive charge, while C60MA, C60ME and C60(OH)_n_ had negative charges with surface potentials of −39.7 ± 1.0 mV, −11.7 ± 0.6 mV, and −29.3 ± 0.6 mV, respectively (**Figure** **7**a). As expected, the surface charge of *Synechocystis* was negative (−24.4 ± 0.3 mV). The surface charge of fullerene derivatives was considered to affect cellular uptake efficiency by changing the electrostatic interaction with the cell surface of *Synechocystis*. Consistently, the *Synechocystis* cells exhibited significant cellular uptake for positively charged C60‐DMePyI and C70‐DMePyI, but no significant uptake for highly negatively charged C60MA and C60(OH)_n_ (Figure 7b; Figures S16 and S17, Supporting Information). Interestingly, a decrease in absorbance for C60ME solution was observed after incubation with *Synechocystis* cells. This phenomenon was possibly resulted from the surface adsorption or co‐sedimentation of *Synechocystis* cells with water‐insoluble C60ME aggregates, especially under strong centrifugal force. Further investigation on the steady‐state fluorescence spectroscopy upon excitation at 570 nm showed that only C60‐DMePyI and C70‐DMePyI caused fluorescence quenching of PSII under *F*_M_ status (Figure S18, Supporting Information). Moreover, fluorescence quenching under *F*_0_ status was also observed in the high‐dose treatments with C60‐DMePyI and C70‐DMePyI (Figure S19, Supporting Information). This further verified the cellular uptake of C60‐DMePyI and C70‐DMePyI, but not C60ME. The absorption spectra of *Synechocystis* in the wavelength range of 350–750 nm suggested that these fullerene derivatives had no significant impact on the photosynthetic active absorption (Figure S20, Supporting Information), indicating their good biocompatibility.

The *Synechocystis* cells treated separately with different fullerene derivatives were used for photocurrent measurement. Among them, only C70‐DMePyI was found to enhance the photocurrent by about three folds, other than C60‐DMePyI (Figure 7c). In fact, a decrease in photocurrent was observed in C60ME‐treated *Synechocystis* cells. This decrease could be attributed to the adherence of C60ME aggregates on the cell surface, which negatively affected cellular viability. The good correlation between photocurrent enhancement and cellular uptake behaviors highlights the importance of surface charge of fullerene derivatives in photosynthesis rewiring. Furthermore, we performed cyclic voltammetry (CV) analysis for these fullerene derivatives in aqueous solutions, the same electrolyte used in photocurrent measurement. Figure 7d shows that there were no distinct oxidation/reduction wave observed for the solutions of C60MA, C60ME and C60(OH)_n_, suggesting these molecules did not exhibit redox activity under experimental conditions. However, two pairs of oxidation/reduction waves were identified in the solutions of C60‐DMePyI and C70‐DMePyI (Figure 7d). This result suggests that both fullerene derivatives can serve as electron carriers, facilitating the connection of photosynthetic electrons between PETCs and the electrode. The relatively higher oxidation/reduction waves of C60‐DMePyI might explain its greater photocurrent enhancement compared to C70‐DMePyI. These results imply that rewiring photosynthesis in PETCs through fullerene molecules was determined by its surface charge and redox properties.

## 3 Conclusion and Outlook

Fullerenes have emerged as attractive 3D electron acceptors and have been widely used in photo‐induced electron transfer, particularly in organic solar cells. The remarkable electronic properties of fullerenes are attributed to its 3D delocalized π systems, which possess large size, spherical strained shape, and high symmetry.^[^ ^36^ ^]^ Besides coupling with organic electron donors, fullerenes have been found to undergo electron exchange with isolated photosynthetic components, including chlorophylls, PSII and PSI, resulting in an enhanced degree of electronic communication.^[^ ^37^, ^38^, ^39^ ^]^ In this study, we demonstrated that a positively charged water‐soluble fullerene derivative can effectively modulate the photosynthetic electron transfer of *Synechocystis*, thereby promoting electron overflow toward the extracellular electrode. This represents the first report of direct rewiring of photosystems in living microorganisms using functional fullerenes for solar‐powered electricity generation, offering a promising strategy for rewiring photosynthesis for diversified applications.

The surface properties of fullerenes play a significant role in determining their cellular uptake and behaviors. While pristine fullerenes like C_60_ and C_70_ have been observed to be taken up by plants through the roots and subsequently translocated to stems and leaves,^[^ ^40^ ^]^ the overall efficiency of plant uptake was generally low due to the hydrophobicity of non‐functionalized fullerenes.^[^ ^41^ ^]^ It was reported that hydroxyl fullerenes were more readily accumulated in plant organs and led to an increase in biomass and fruit yield.^[^ ^42^ ^]^ In this work, a hydrophilic group was introduced to the surface of the fullerene cage, imparting water solubility to these nanoparticles and enabling good dispersion in aqueous solutions. It was demonstrated that the positive charge of C60‐DMePyI is required for efficient cellular uptake, indicating electrostatic binding between fullerene derivatives and the negatively charged surface of *Synechocystis* cells. The intrinsic hydrophobic properties of the fullerene cage might facilitate the translocation of fullerene molecules through the lipid bilayer of cell membranes.^[^ ^43^ ^]^

Redirecting electron transfer by interfering with its original pathways can be challenging since the redox centers are typically embedded inside the interior of complex photosystems. However, the unique cage‐like structure and electron‐deficient nature enabled fullerenes to capture photosynthetic electrons from various redox sites in PETCs, including redox centers of PSII and PSI. Recently, it has been reported that several small‐molecule mediators, such as DCBQ, can directly extract electrons from the excited reaction centers of photosystems.^[^ ^35^ ^]^ These findings open up possibilities for rewiring photosynthesis to achieve theoretical maximum electron harvesting for applications in man‐made systems. Besides extracting more electrons from photosynthesis, fullerenes possess the ability to facilitate electron transfer across natural cell membranes by incorporating themselves within the lipid bilayers, as demonstrated in artificial liposomes.^[^ ^44^ ^]^ We envision the practical development of a bio‐solar cell that combines the high charge separation efficiency of cyanobacteria with the strong electron extraction capacity of fullerenes.

## 4 Experimental Section

### Materials

Fullerenes C_60_ (99.9%, Aladdin), C_60_‐*N,N*‐dimethyl pyrrolidinium iodide (>99%, Solaris Chem), C_70_‐*N,N*‐dimethyl pyrrolidinium iodide (>99%, Solaris Chem), C_60_‐malonic acid (>99%, Solaris Chem), C_60_‐malonic ester (>99%, Solaris Chem), fullerenol (99.9%, Aladdin), 3‐(3,4‐dichlorophenyl)−1,1‐dimethyl urea (99%, Aladdin), 2,5‐dibromo‐3‐methyl‐6‐isopropylbenzoquinone (Sigma‐Aldrich), phenylmercuric acetate (Hampton Research) and 2‐heptyl‐4‐hydroxy‐quinoline‐*N*‐oxide (Apexbio) were purchased from commercial suppliers and used without further purification unless otherwise noted. The fullerene derivatives were suspended in deionized water at a concentration of 10 mg mL^−1^, followed by sonication for 30 min before use. The stock solutions of inhibitors including DCMU, DBMIB, PMA, and HQNO were prepared in ethanol.

### Culture Conditions

The wild‐type strain of *Synechocystis* sp. PCC 6803 and *Synechococcus elongatus* PCC 7942 were grown in BG11 medium at 30 °C under 3% CO_2_ and continuous light of 150 µmol photons m^−2^ s^−1^. The inoculation cell density was set as 0.2, and 50 mm NaHCO_3_ was added as the initial carbon source. The *Synechocystis* and *Synechococcus* cells grown for three days were collected for photocurrent measurement and other experiments.

### Characterization of Fullerenes

The chemical composition of fullerene C_60_ and its derivative C60‐DMePyI were characterized by XPS (ESCALAB 250Xi, Thermo Fisher Scientific, USA) and FTIR (Nicolet 6700, Thermo Fisher Scientific, USA). The ultrathin carbon films‐supported C60‐DMePyI nanoparticles were observed by TEM (Tecnai G20, FEI Co., USA), and their size was determined using the Nano Measurer (version 1.2.5). The cellular distribution of C60‐DMePyI was examined by TEM (JEM‐1200EX, JEOL, Japan), using the cross‐sectional slices of *Synechocystis* cells. The zeta potentials of fullerene derivatives dispersed in deionized water were measured using Zetasizer Nano ZS (Malvern Panalytical Ltd, UK).

### Internalization of Fullerenes by *Synechocystis*

The *Synechocystis* cells grown for 3 days were harvested by centrifugation at 12 000× g for 2 min. The supernatant was removed and the precipitated pellet was resuspended in fresh BG11 medium free of ammonium ferric citrate. The optical density at 730 nm (OD_730_) of the concentrated *Synechocystis* cells was adjusted to 25. Unless otherwise stated, the fullerene derivatives were added to the concentrated *Synechocystis* cells at a final concentration of 0.1 mg mL^−1^. The mixture of cells and nanomaterials was incubated under a low light condition (≈30 µmol photons m^−2^ s^−1^) for 2 h to allow for interaction between nanomaterials and cells. To determine the cellular uptake efficiency, the absorption spectra of supernatants collected at different times were recorded between 230 and 750 nm using a spectrophotometer (TU‐1900, Persee, China). The absorption spectra of *Synechocystis* cells were measured between 380 and 750 nm.

### Electrochemical Experiments

Photocurrent measurements were performed in a three‐electrode electrochemical system using ITO conductive glass as the working electrode, platinum wire as the counter electrode, and Ag/AgCl as the reference electrode. The BG11 medium free of ammonium ferric citrate was used as the electrolyte. After incubation with fullerene derivatives, 200 µL of cyanobacteria‐nanomaterial mixture was dropped onto a clean ITO conductive glass and allowed to dry at room temperature. For inhibitor experiments, a prepared stock solution of a specific inhibitor was added to the cyanobacteria‐nanomaterial mixture before dropping it. An equal volume of ethanol was added in control groups. Once dried, the electrolyte was immediately added to the setup to prevent cell death. The geometrical surface area of formed biofilm was 2.1 cm^2^ and the final volume of the electrolyte solution was 20 mL. The *Synechocystis* biofilms were illuminated using a monochromatic red light source (*λ* = 658 nm) with an intensity of 400 µmol photons m^−2^ s^−1^, unless otherwise stated.

Stepped chronoamperometry scan were conducted on *Synechocystis* biofilms to analyze the photoresponse profiles as a function of the applied potential. The applied potential of the working electrode was controlled using a potentiostat (CHI1030C, CH Instruments, China). Each potential was held for 250 s before being stepped up by 0.1 V. During this period, the working electrode was allowed to equilibrate for 100 s in the dark, followed by irradiation for 100 s and then light off for 50 s. Dark current spikes caused by potential stepping were excluded from all figures for clarity. Photocurrent measurements were conducted under chopped light illumination (100 s light/100 s dark cycles) at a constant applied potential of 0.8 V versus Ag/AgCl. CV studies of fullerene derivatives were performed on screen‐printed gold electrodes (250AT) using a portable potentiostat (µStat‐i 400, DropSens, Spain) with DropView 8400 operating software at a scan rate of 50 mV s^−1^. An aqueous solution of fullerene derivatives with 0.1 mg mL^−1^ was directly dropped on the screen‐printed electrode, covering all three electrodes for CV experiments.

### PAM Fluorometry

Photochemical efficiencies in PSII and PSI were determined by measuring chlorophyll fluorescence using PAM fluorometry and saturation pulse technology. After incubation with C60‐DMePyI, the cyanobacterial suspension with an OD_730_ of 5.0 was transferred into a quartz cuvette with 1 cm path length. Slow kinetics and light curve were recorded by Dual‐PAM‐100 fluorometer (Walz, Germany) for obtaining the kinetic parameters of chlorophyll fluorescence. Three complementary quantum yields Y(II), Y(NO), Y(NPQ) for PSII, and Y(I), Y(ND), Y(NA) for PSI were analyzed by Dual‐PAM software. Chlorophyll fluorescence transient was measured using Dual‐PAM‐100 fluorometer with the fast acquisition mode. Prior to measurement, all samples were dark‐adapted for 30 min. The fluorescence transient data were analyzed using the JIP‐test.^[^ ^45^ ^]^ The fluorescence intensities at 50 µs (O‐step), 2 ms (J‐step), 30 ms (I‐step) and the maximal fluorescence (P‐step) were denoted as F_0_, F_J_, F_I_, and F_M_, respectively. The selected JIP‐test parameters that quantify PSII behavior and the corresponding values calculated from the original data are listed in Table S1 (Supporting Information).

### Steady‐State Fluorescence Spectroscopy

Fluorescence emission spectra were recorded using the F‐7000 fluorescence spectrophotometer (Hitachi, Japan). The excitation wavelength was set at 435, 475, and 570 nm for preferentially exciting chlorophyll *a*, chlorophyll *b*, and phycobilisomes, respectively. The emission spectra between 600 and 800 nm of *Synechocystis* were then recorded. Prior to measurement, the OD_730_ of cell suspensions was adjusted to 1.0. The *F*_M_ status of cyanobacterial cells was attained by the addition of DCMU at a saturating concentration of 0.2 mm and treated in room temperature for half an hour.

### TA Spectroscopy

TA spectroscopy was carried out by using a Helios pump‐probe system (Ultrafast Systems) combined with a regenerative‐amplified Ti: sapphire laser system (Legend Elite‐1K‐HE, center wavelength of 800 nm, pulse width of 25 fs, pulse energy of 4 mJ, repetition rate of 1 kHz) and an optical parametric amplifier system. The output light of regenerative‐amplified Ti: sapphire laser (800 nm) is split into two beams. The main part of the fundamental beam (800 nm) was sent to the optical parametric amplifiers (TOPAS‐C), which generated a pump pulse with tunable wavelength from 290 nm to 2.6 µm. The intensity of the pump pulse used in the experiment was controlled by a variable neutral‐density filter wheel. A small part of the fundamental beam (800 nm) was introduced into the TA spectrometer in order to generate the probe light. After passing through a motorized optical delay line, the 800‐nm light beam was attenuated with a neutral density filter and focused on a sapphire crystal, which was used to generate the white‐light continuum (WLC) probe pulses with wavelength of 460 to 820 nm, respectively. The probe beam and the pump beam were focused and overlapped onto the sample. The optical path difference between the pump light and the probe light, which was controlled by the motorized optical delay‐line, was used to get the relative time delay between the probe light and the pump light. After sampling, the probe beam was collimated and then focused into a fiber‐coupled spectrometer and detected at a frequency of 1 KHz. Chopped by a synchronized chopper at frequency of 500 Hz, the pump pulse was modulated such that the TA spectra with and without the pump pulses could be recorded alternately. The pump pulse was set to 440 nm. The sample solutions at an OD_730_ of 2.0 were placed in cuvettes with 1 mm path length. One transient measurement takes ≈20 min and includes three individual scans. Data analysis was carried out using Surface Xplorer software.

### Cytotoxicity Assays

The *Synechocystis* cells were incubated with different concentrations of fullerene derivative C60‐DMePyI for 12 h. Following incubation, the cells were resuspended in fresh BG11 and the concentration was adjusted to an OD_730_ of 1.0. Aliquots (10 µl) of serial dilutions (10^0^, 10^−1^, 10^−2^, 10^−3^) were spotted on BG11 agar plates, which were cultured at 30 °C and 50 µmol photons m^−2^ s^−1^ light for one week. Cell membrane integrity was evaluated by fluorescence staining using PI. The stain solution was prepared by adding 3 µL of 20 mm PI in DMSO to 2 mL BG11 medium. The 100 µl aliquots of stain solutions were added to the 100 µl cell suspensions in 96‐well black plates. The microplate was incubated at room temperature in the dark for 15 min. Fluorescence intensity was measured at an excitation wavelength of 485 nm and an emission wavelength of 630 nm. The dead cells treated by adding 70% isopropanol for half an hour was used as positive control.

### Ferredoxin Redox Assay

The coding gene (ssl0020) of ferredoxin from *Synechocystis* was cloned into the expression plasmid pET‐28a with an N‐terminal 6×His‐tag fusion. The recombinant plasmid was transformed into *E. coli* BL21(DE3). For protein expression, *E. coli* strain was grown at 37 °C in 100 mL Luria‐Bertani (LB) medium supplemented with 0.5 mm FeCl_3_ and 1 mm cysteine. When an OD_600_ of 0.6–1.0 was reached, the culture was cooled to 18 °C and induced by the addition of 0.2 mm isopropyl‐β‐d‐thiogalactopyranoside (IPTG). After culture for 18–20 h, the cells were harvested by centrifugation at 8000 g for 5 min and resuspended in 20 mm Tris‐HCl buffer, pH 8.0, containing 20 mm MgCl_2_. The cell lysate by sonication was purified by Ni‐NTA resin columns according general procedures. The protein concentration was determined by the Bradford method. The purity of ferredoxin was analyzed by SDS‐PAGE. The purified ferredoxin was stored at −20 °C by adding 10% glycerol. The redox reactions of purified ferredoxin were performed under anaerobic conditions in 20 mm Tris‐HCl buffer, pH 8.0, containing 75 mm NaCl. The purified ferredoxin in oxidation state (brown color) was reduced by the addition of tenfold excess of sodium dithionite solution. The dithionite‐reduced ferredoxin was reoxidized by adding fullerene C60‐DMePyI solution. The absorption spectra of ferredoxin suspension in different states were measured in a quartz cuvette sealed with a silicone rubber stopper.

### Statistical Analysis

The data were presented as mean values ± standard deviation (SD) based on experiments performed in triplicate or more. Statistical data analysis was performed using unpaired two‐tailed Student's *t*‐test in GraphPad Prism. Statistically significant differences were represented with *p < 0.05, **p < 0.01, and ***p < 0.001.

## Conflict of Interest

The authors declare no conflict of interest.

## Author Contributions

H.Z., T.H., and Y.L. conceived the project. H.Z. and J.L. conducted the TA spectroscopy. H.Z. conducted all other experiments. H.Z., F.M.C., T.H., and Y.L. analyzed the experimental data. H.Z. and Y.L. wrote the manuscript. All authors critically revised and approved the final manuscript.

## Supporting information

Supporting Information

## Data Availability

The data that support the findings of this study are available from the corresponding author upon reasonable request.
