# Supplementary material for: Rewiring Photosynthesis by Water‐Soluble Fullerene Derivatives for Solar‐Powered Electricity Generation
Source: Adv Sci (Weinh). 2024 Apr 22;11(23):2310245. doi: 10.1002/advs.202310245 (PMC11187915; doi:10.1002/advs.202310245)
Supplement: Supplementary file 1 — Supporting Information [file ADVS-11-2310245-s001.pdf]

ADVANCED SCIENCE
Open Access

Supporting Information

for *Adv. Sci.*, DOI 10.1002/advs.202310245

Rewiring Photosynthesis by Water-Soluble Fullerene Derivatives for Solar-Powered Electricity Generation

Huawei Zhu, Franco M. Cabrerizo, Jing Li, Tao He* and Yin Li**

Supporting Information

Rewiring Photosynthesis by Water-Soluble Fullerene Derivatives for Solar-Powered Electricity Generation

*Huawei Zhu**, *Franco M. Cabrerizo, Jing Li, Tao He**, *and Yin Li**

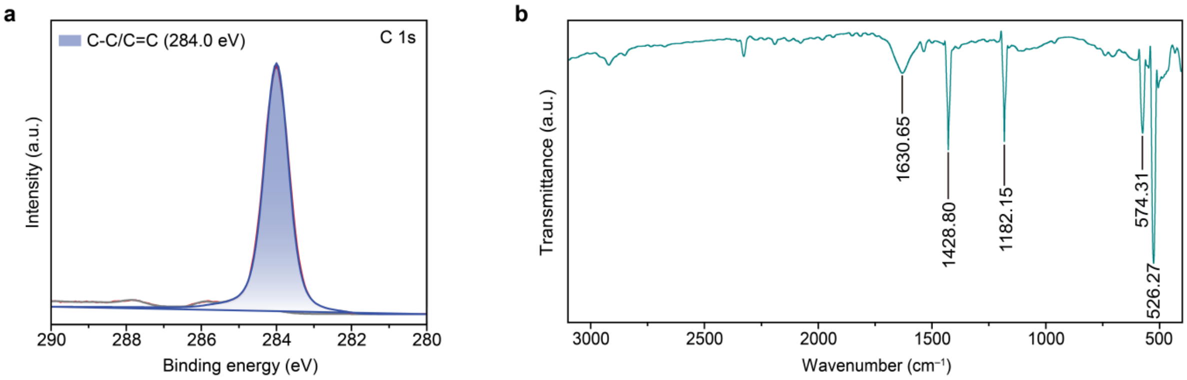

Figure S1. Characterization of pristine fullerene C_{60}. a) XPS spectrum of C_{60}. b) FTIR spectrum of C_{60}.

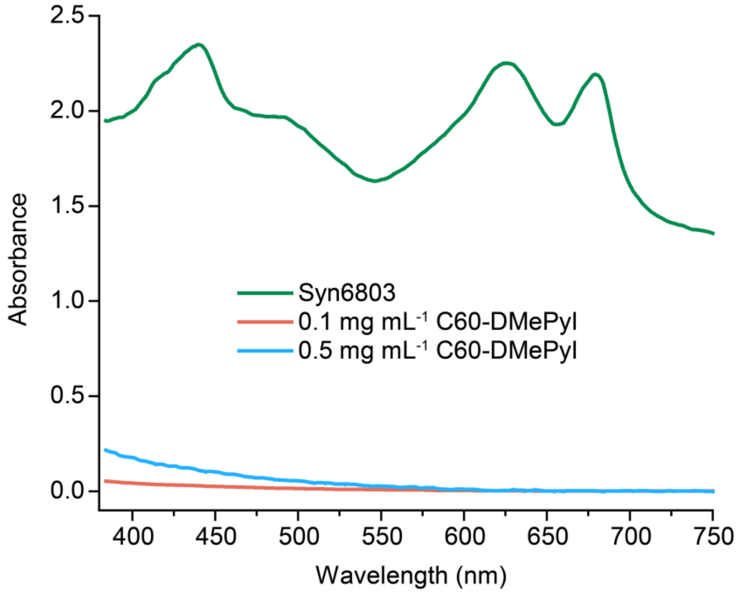

Figure S2. The absorption spectra of *Synechocystis* sp. PCC 6803 and C60-DMePyI aqueous solutions. The concentration proportions of *Synechocystis* and C60-DMePyI corresponds to those in incubation mixtures used for photocurrent measurement.

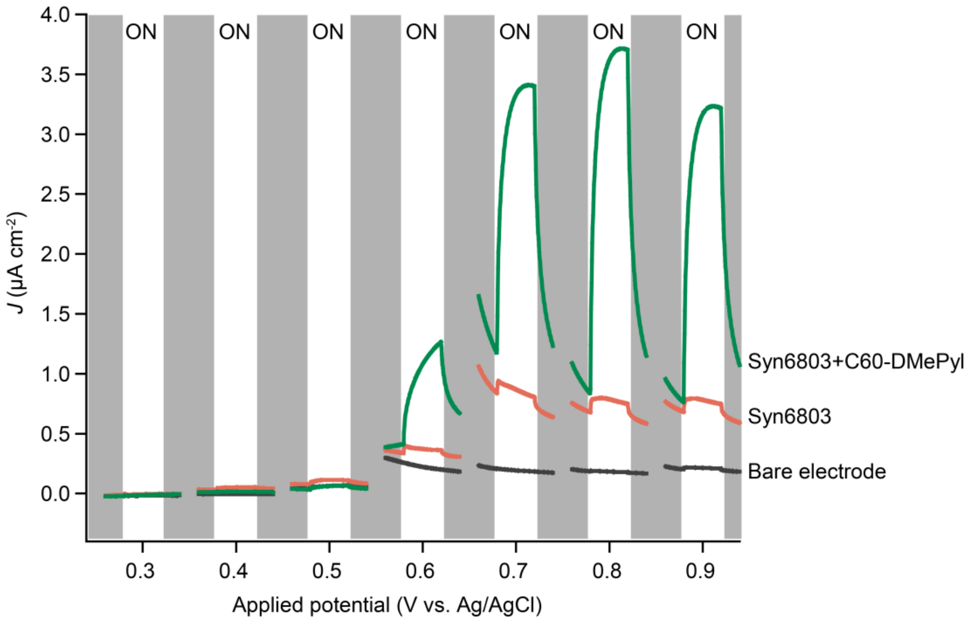

Figure S3. Stepped chronoamperometry scans on *Synechocystis* biofilms under chopped light irradiation. The photoresponses profiles of bare electrode (ITO glass), *Synechocystis* alone and *Synechocystis* treated with C60-DMePyI were recorded under different applied potentials of working electrode. The gray columns indicate light off. A monochromatic red light source (λ = 658 nm) with an intensity of 400 μmol photons m^{-2} s^{-1} was used.

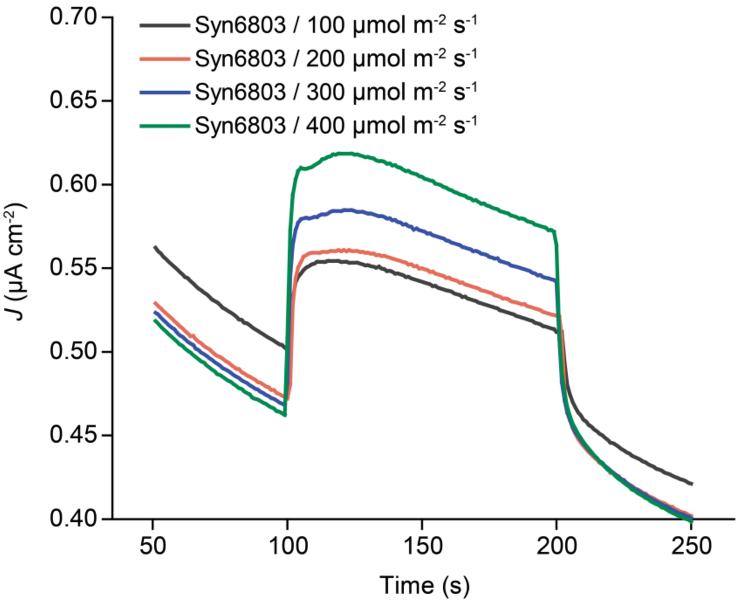

Figure S4. The photocurrents generated by *Synechocystis* cells alone under different light intensities.

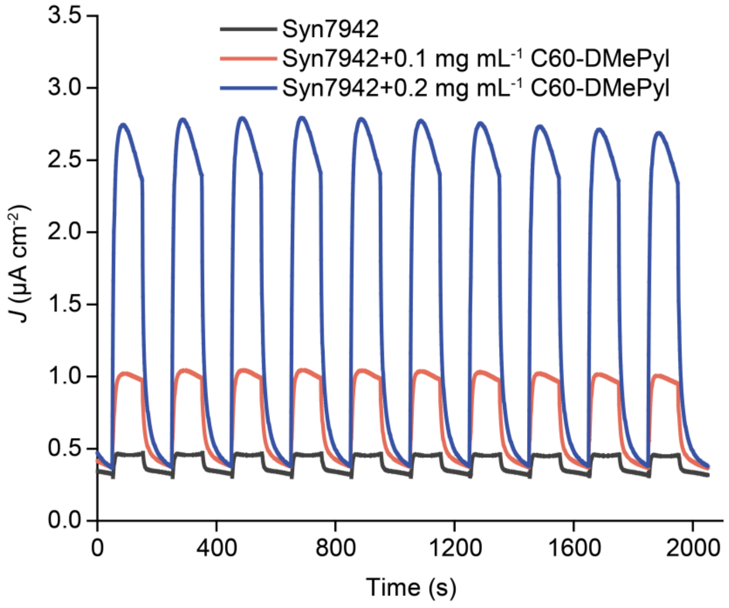

Figure S5. Enhanced photocurrent generation of *Synechococcus elongatus* PCC 7942 by C60-DMePyI rewiring.

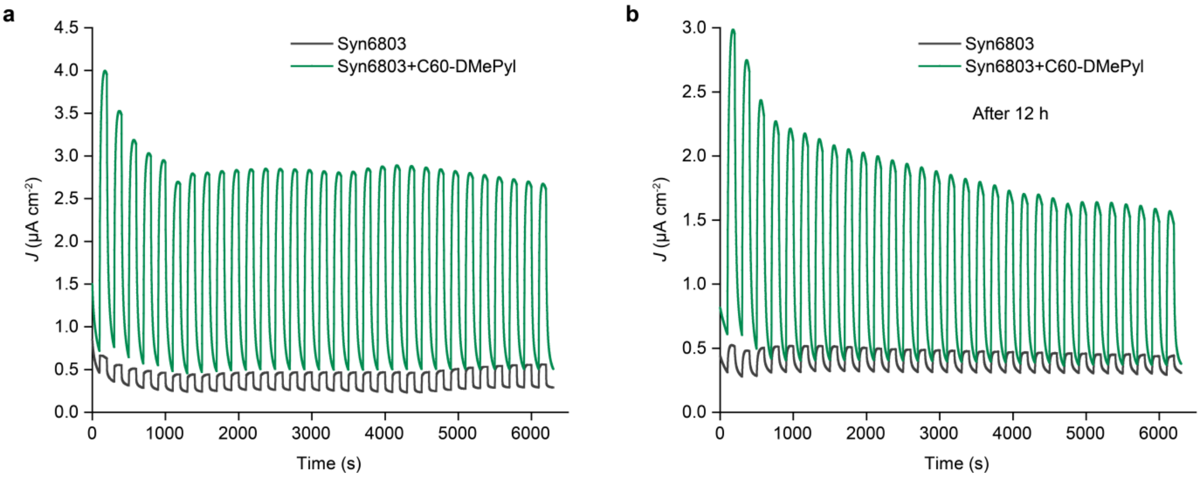

Figure S6. Long-term performance of photocurrent generation. a) Photocurrent generation over thirty light/dark cycles (~2 h). b) Successive photocurrent generation over thirty light/dark cycles after kept the electrochemical devices at room temperature for 12 h.

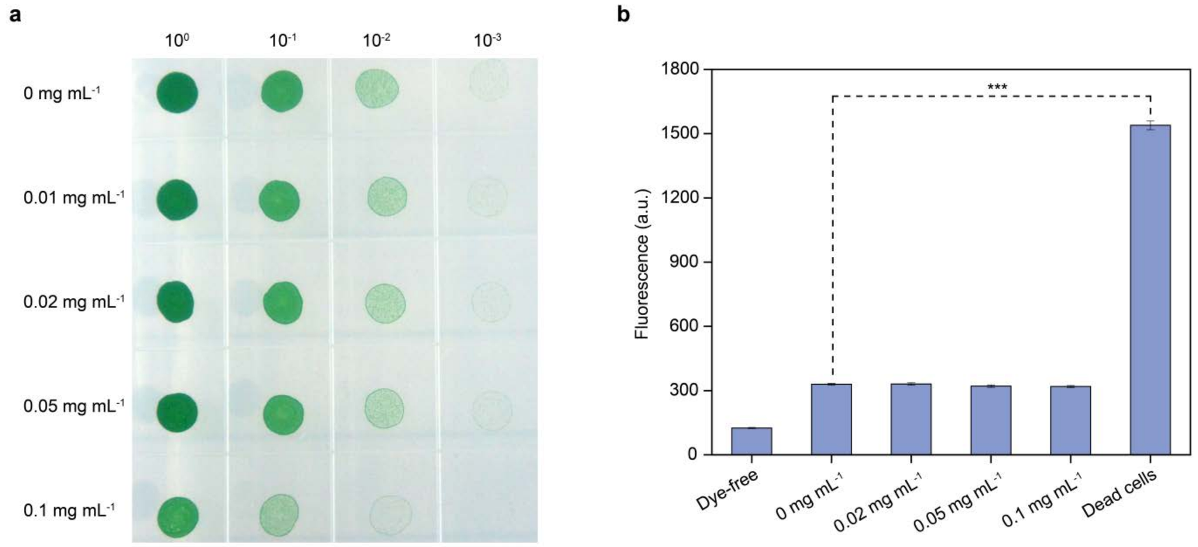

Figure S7. Cytotoxicity assays of C60-DMePyI. a) Cytotoxicity spot assay. *Synechocystis* cells were incubated with C60-DMePyI at different concentrations for 12 h. Serial dilutions were prepared, spotted on BG11 agar plate and cultured for one week. b) Fluorescence intensity of PI stained *Synechocystis* cells that treated with C60-DMePyI at different concentrations. The dead cells that treated with 70% isopropanol for half an hour was used as positive control. The excitation and emission wavelengths were set at 485 nm and 630 nm, respectively. The data are presented as mean values ± SD from independent biological replicates (n = 3). Statistical significance was analyzed using unpaired two-tailed Student's t-test, and significance levels were denoted as $*p < 0.05$, $**p < 0.01$, $***p < 0.001$.

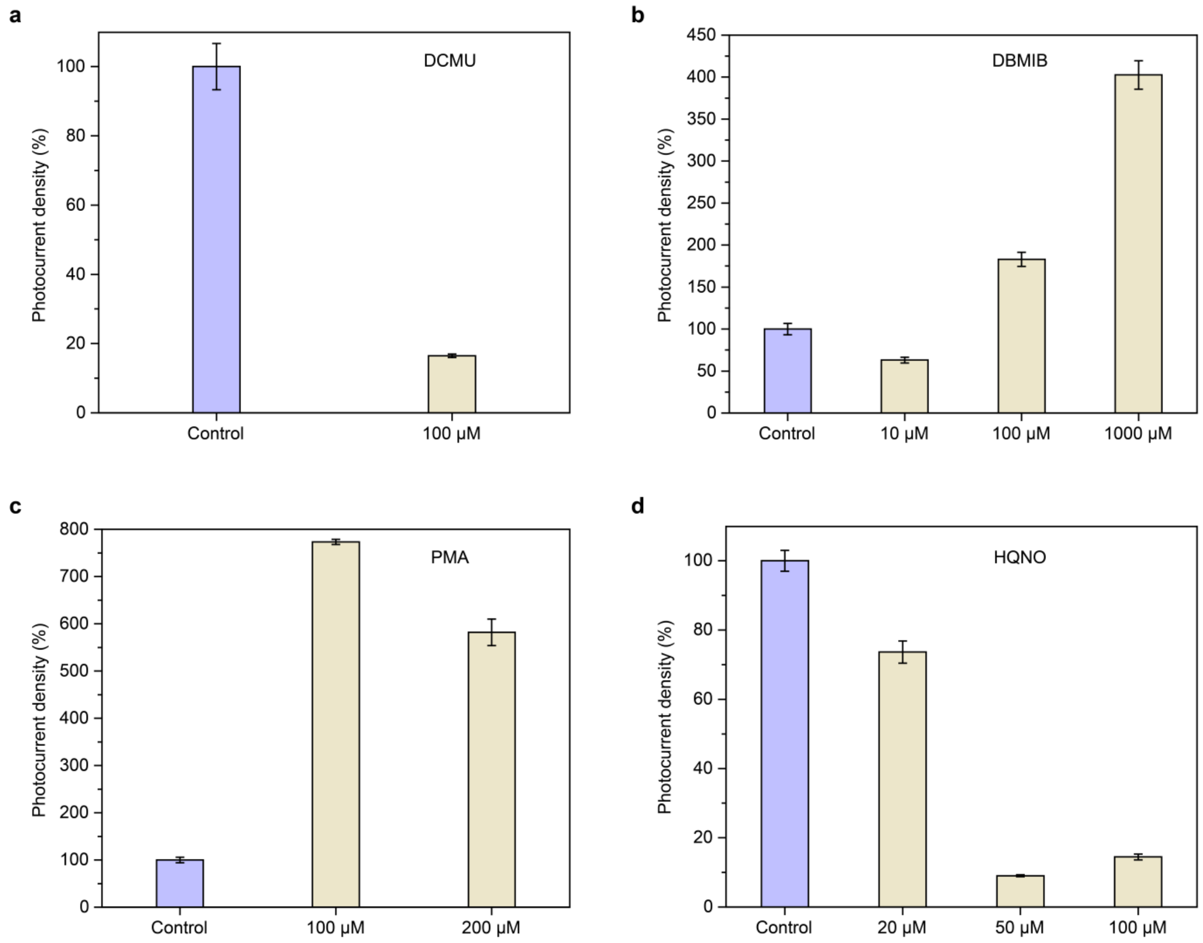

Figure S8. The effects of site-specific inhibitors on photocurrent generation of *Synechocystis* alone. a) The photocurrent generation under DCMU inhibition. b) The photocurrent generation under DBMIB inhibition. c) The photocurrent generation under PMA inhibition. d) The photocurrent generation under HQNO inhibition.

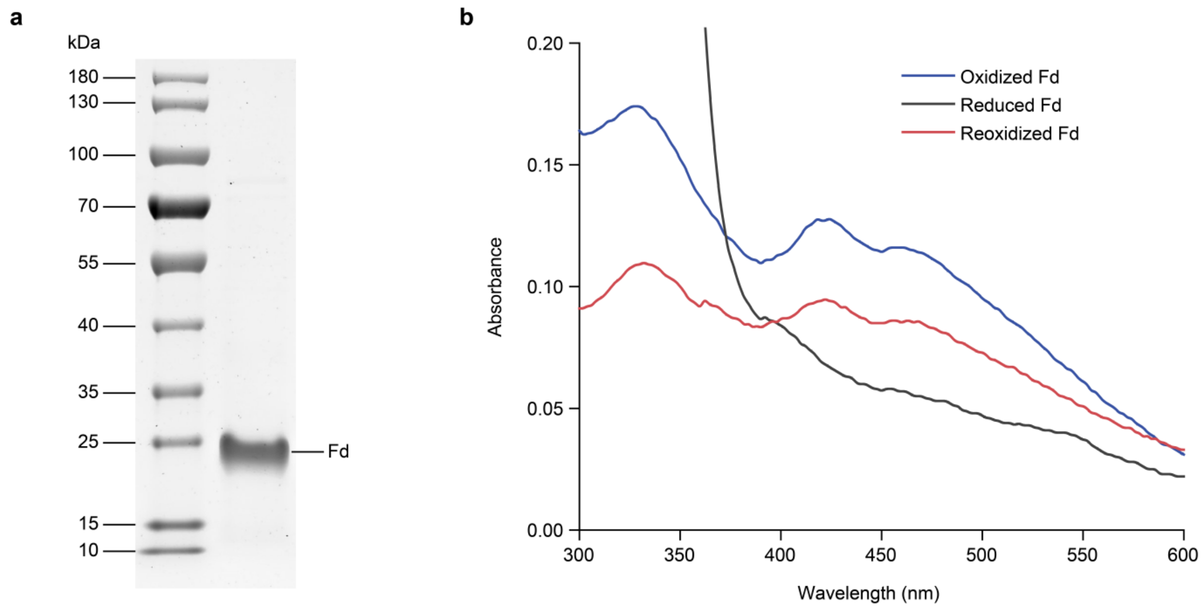

Figure S9. Ferredoxin redox assays. a) SDS-PAGE of purified *Synechocystis* ferredoxin heterologously expressed in *E. coli* BL21(DE3). b) In vitro re-oxidation of dithionite-reduced ferredoxin by C60-DMePyI under anaerobic conditions. The UV-visible absorption spectra of ferredoxin at different states were recorded. The oxidized ferredoxin shows the characteristic absorption peaks at near 330 nm, 420 nm and 460 nm.

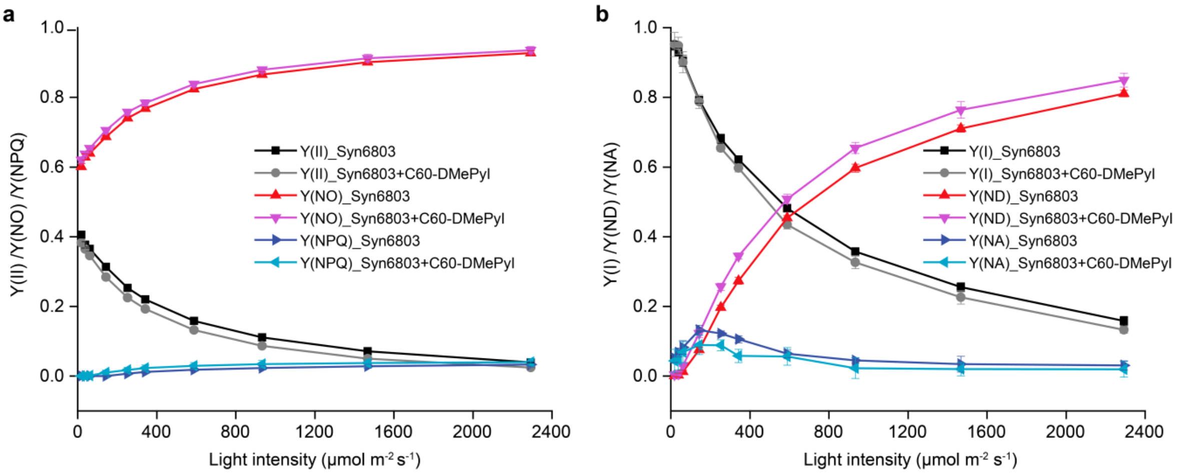

Figure S10. The effects of C60-DMePyI on the photochemical quantum efficiencies of PSII and PSI. a) The complementary quantum yields of PSII, including Y(II), Y(NO) and Y(NPQ). Y(II) represents the effective photochemical quantum yield of PSII, Y(NO) represents the quantum yield of non-light-induced non-photochemical fluorescence quenching and Y(NPQ) represents the quantum yield of light-induced non-photochemical fluorescence quenching. b) The complementary quantum yields of PSI, including Y(I), Y(ND) and Y(NA). Y(I) represents the effective photochemical quantum yield of PSI, Y(ND) represents the quantum yield of non-photochemical energy dissipation due to donor side limitation and Y(NA) represents the quantum yield of non-photochemical energy dissipation due to acceptor side limitation. The data are presented as mean values ± SD from independent biological replicates (*n* = 3).

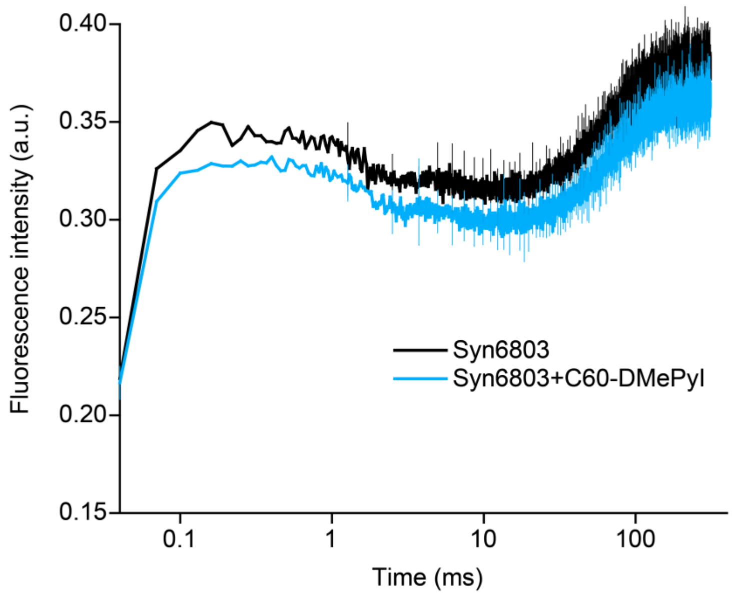

Figure S11. The OJIP fluorescence transients of *Synechocystis* alone and the *Synechocystis* treated with C60-DMePyI. The data are presented as mean values ± SD from independent biological replicates (n = 10).

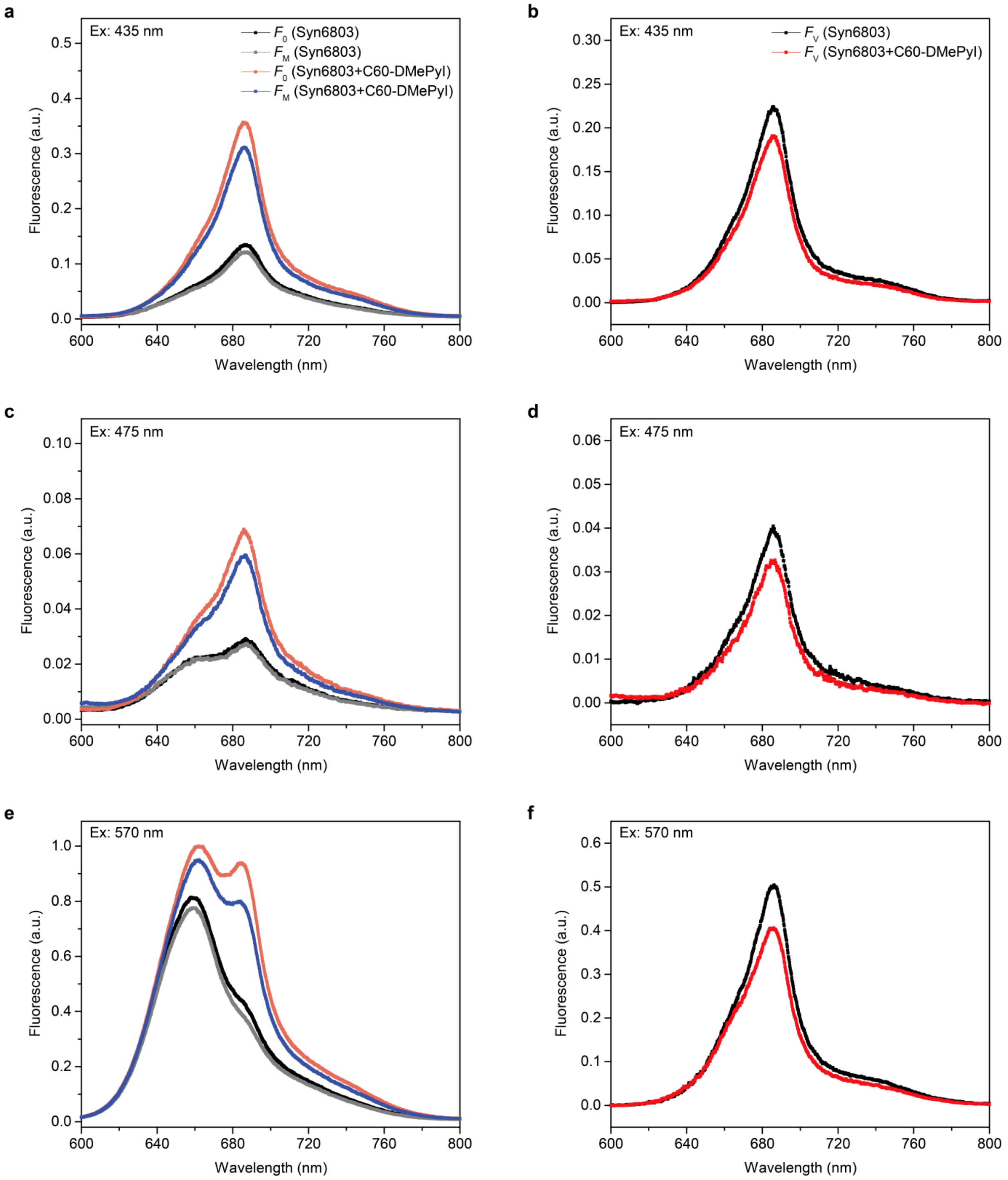

Figure S12. Fluorescence emission spectra of *Synechocystis* recorded under open and closed PSII reaction centers. Minimal fluorescence emission (F_0) under open reaction centers and maximal fluorescence emission (F_M) under closed reaction centers were recorded at the excitation wavelength of 435 nm (a), 475 nm (c) and 570 nm (e). Variable fluorescence (F_V = F_M-F_0) for excitation at 435 nm (b), 475 nm (d) and 570 nm (f) were calculated accordingly. The spectra were normalized between 0 and 1. The concentration of C60-DMePyI was 0.1 mg mL^{-1}.

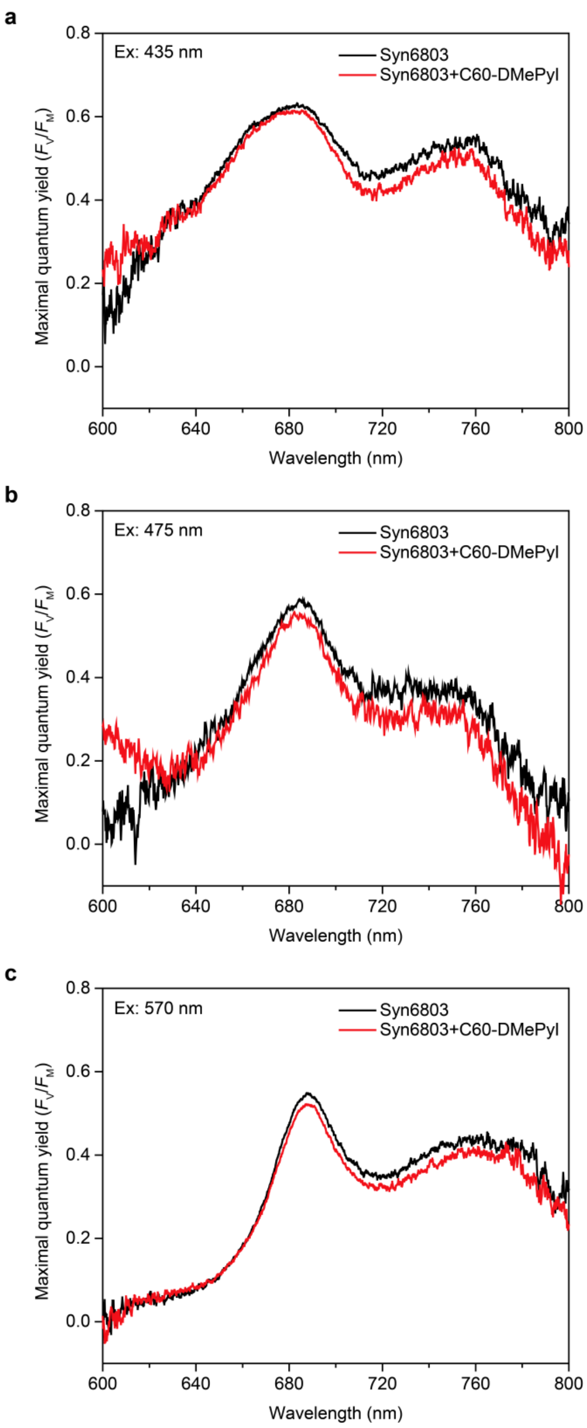

Figure S13. Maximum quantum yield (F_V/F_M) for excitation at 435 nm (a), 475 nm (b) and 570 nm (c), derived from fluorescence emission spectra in Fig S6. The concentration of C60-DMePyI was 0.1 mg mL^{-1}.

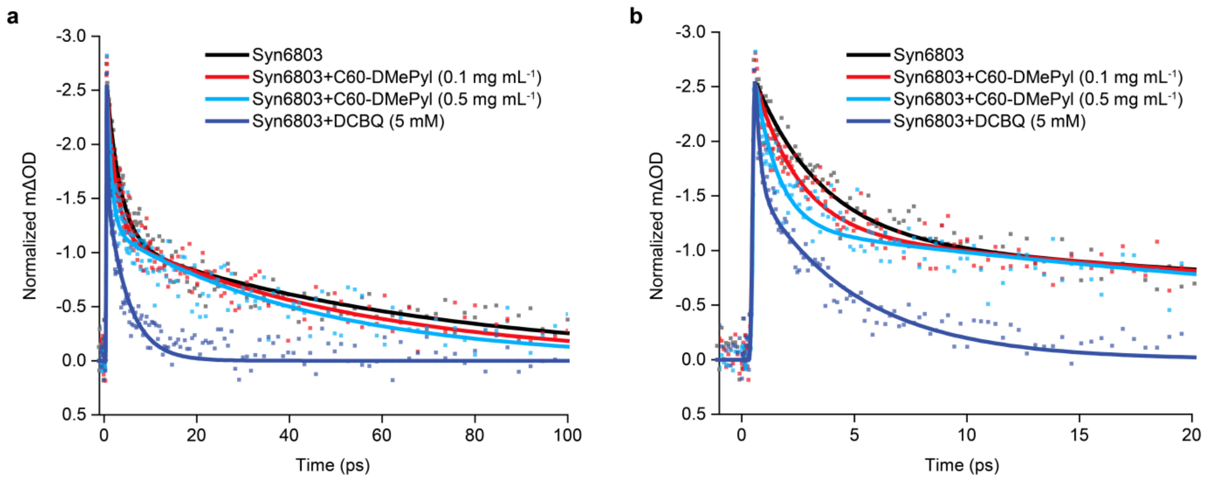

Figure S14. Transient absorption kinetics of *Synechocystis* at 690 nm. a) Transient absorption kinetics at time scale of -2 to 100 ps. b) Transient absorption kinetics at time scale of -2 to 20 ps. Square scatters represent original data and solid lines indicate fit curves (bi-exponential). The fitted kinetic parameters were summarized in Table S2.

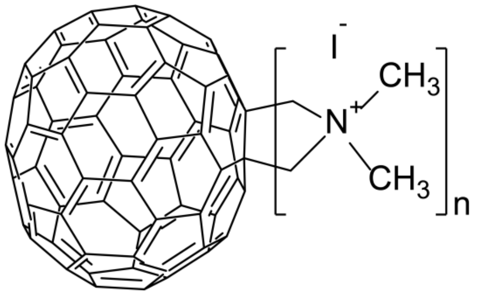

C70-DMePyI
(C_{70}-*N*,*N*-dimethyl pyrrolidinium iodide)

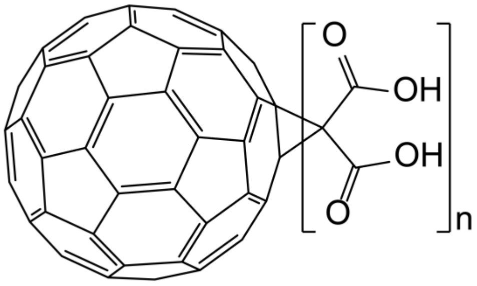

C60MA
(C_{60}-malonic acid)

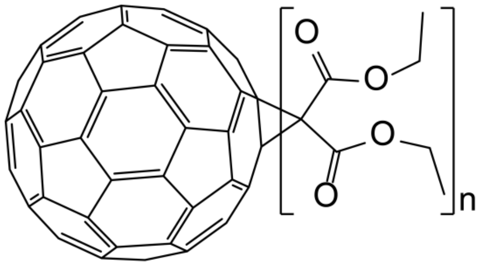

C60ME
(C_{60}-malonic ester)

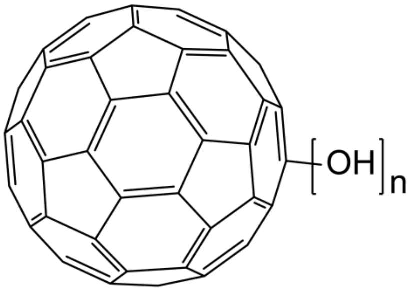

C60(OH)$_n$
(Fullerenol/polyhydroxylated C_{60})

Figure S15. Chemical structures of different functionalized fullerene derivatives.

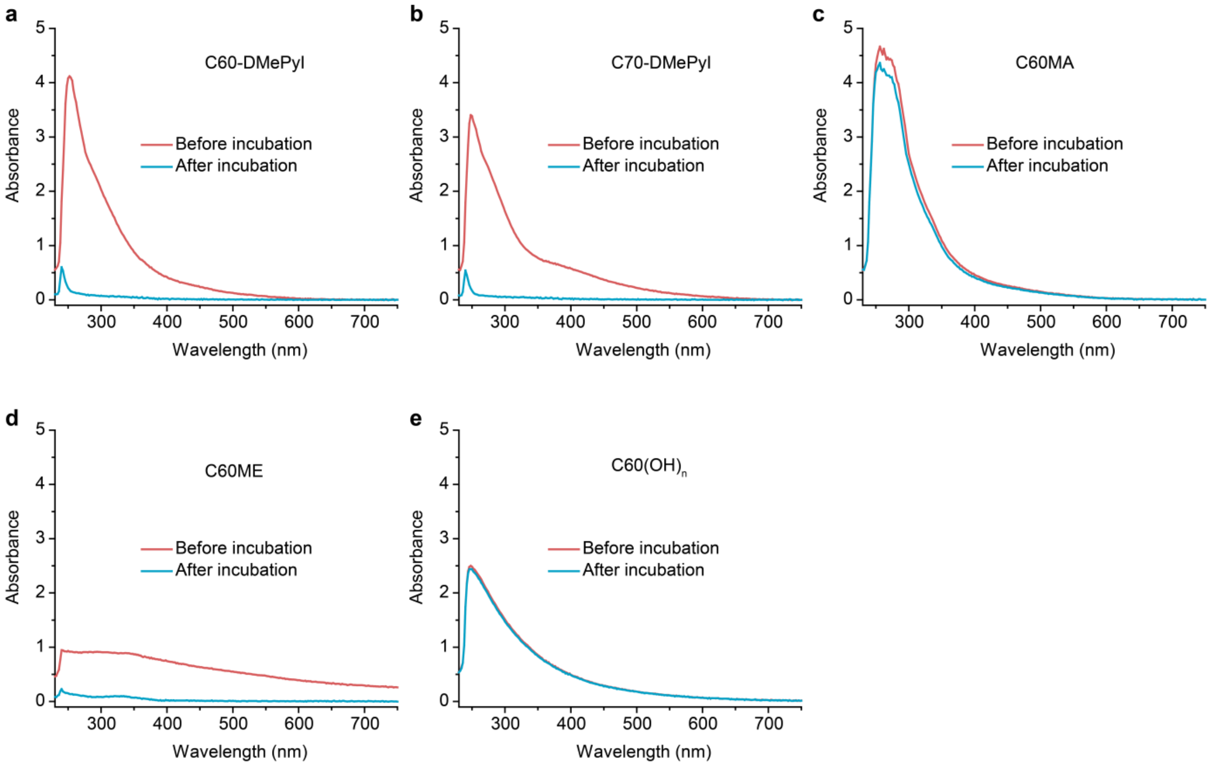

Figure S16. The absorption spectra of aqueous solutions of different fullerene derivatives before and after incubation with *Synechocystis* cells. a) C60-DMePyI. b) C70-DMePyI. c) C60MA. d) C60ME. e) C60(OH)$_n$.

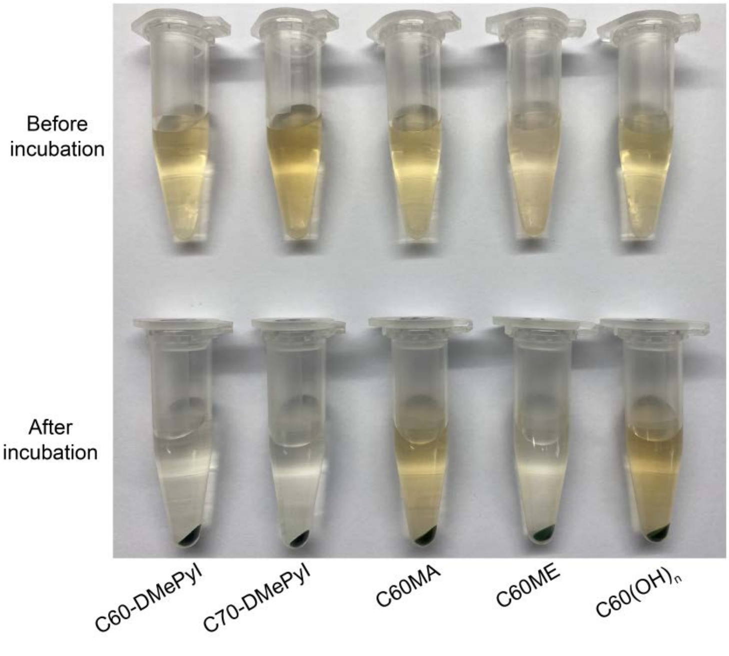

Figure S17. The photographs of aqueous solutions of different fullerene derivatives before and after incubation with *Synechocystis* cells.

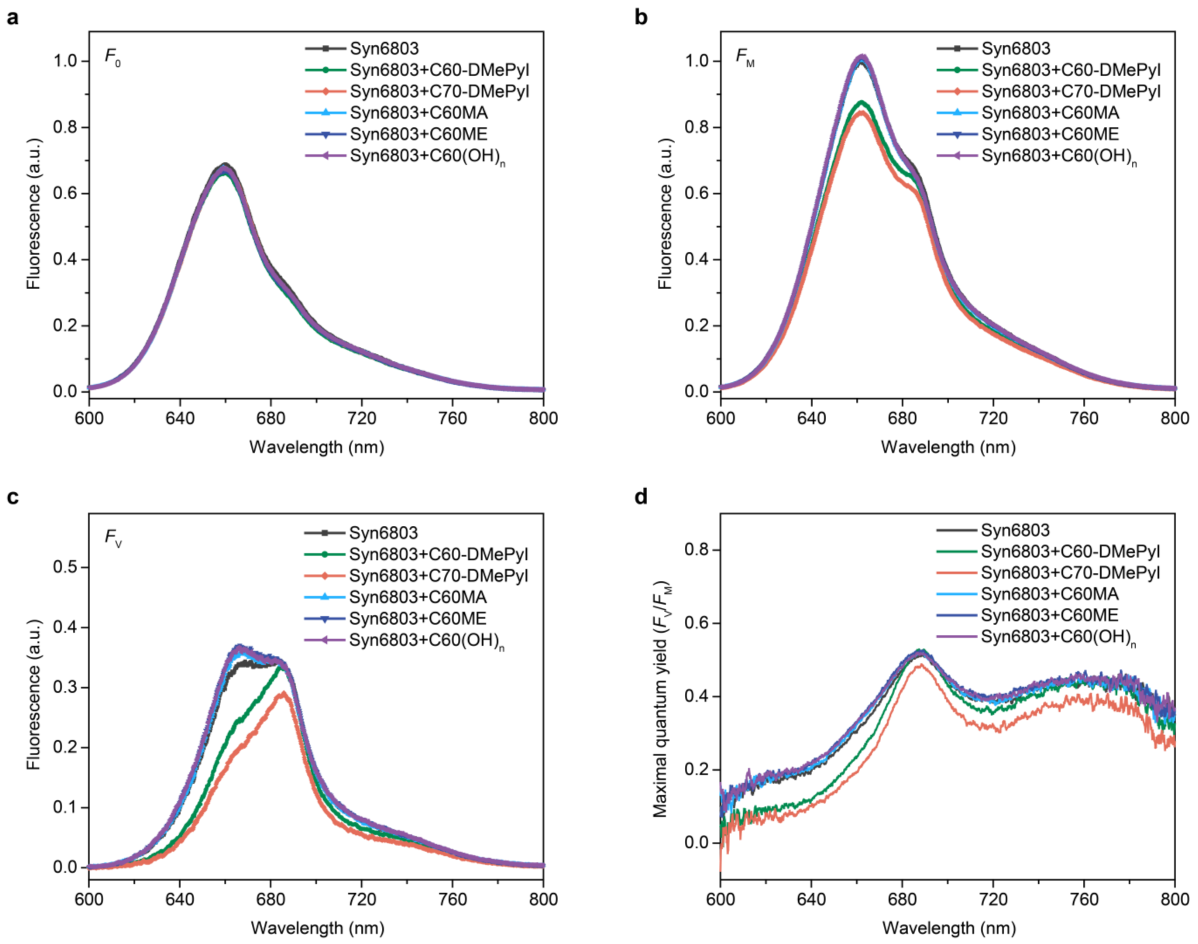

Figure S18. Fluorescence emission spectra of *Synechocystis* after incubated with different fullerene derivatives. a) Minimal fluorescence emission (F_0) recorded under open PSII reaction centers. b) Maximal fluorescence emission (F_M) recorded under closed PSII reaction centers. c) Variable fluorescence ($F_V = F_M$-F_0). d) Maximum quantum yield (F_V/F_M). The excitation wavelength was set at 570 nm and the spectra were normalized between 0 and 1. The concentration of fullerene derivatives was 0.1 mg mL^{-1}.

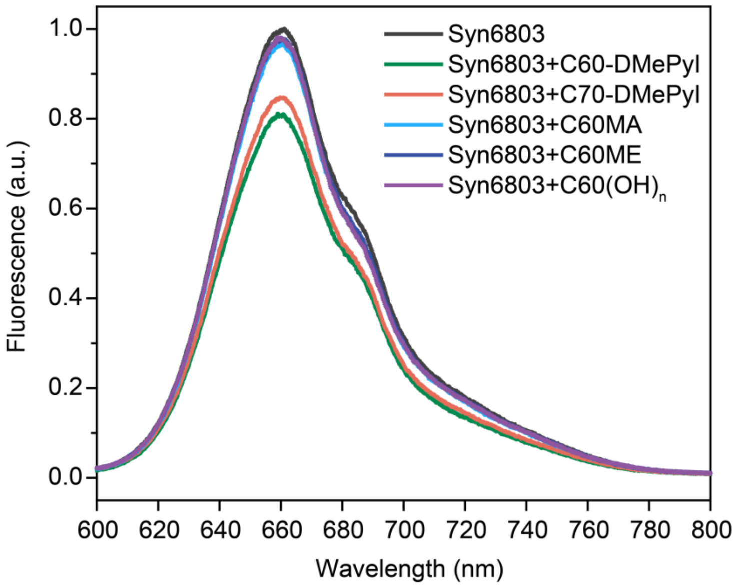

Figure S19. Fluorescence emission spectra of *Synechocystis* treated with high-dose fullerene derivatives. The *Synechocystis* cells were treated with different fullerene derivatives at a concentration of 1.0 mg mL^{-1} for half an hour. The residual fullerene derivatives in the supernatants were washed out before measurements. The fluorescence emission was recorded under open PSII reaction centers (F_0) at an excitation wavelength of 570 nm.

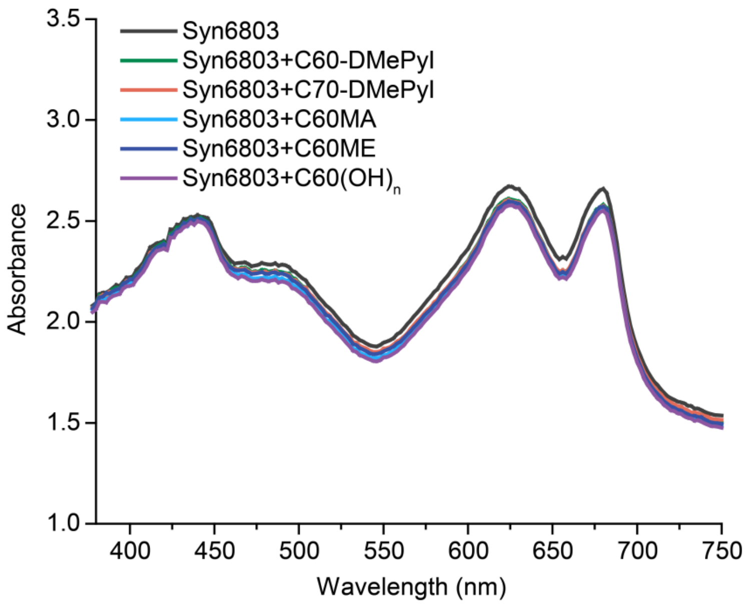

Figure S20. The absorption spectra of *Synechocystis* cells after incubated with different fullerene derivatives at a concentration of 1.0 mg mL^{-1}.

Table S1. Summary of the JIP-test for OJIP fluorescence transients.

Parameters	Terms	Syn6803	Syn6803 +C60-DMePyI	Significantly different (p <0.05)
**Technical parameters**				
F_0	Minimal fluorescence intensity at 50 μs	0.219 ± 0.010	0.217 ± 0.008	No
F_{300}	Fluorescence intensity at 300 μs	0.343 ± 0.017	0.329 ± 0.012	No
F_J	Fluorescence intensity at J-step (2 ms)	0.328 ± 0.014	0.308 ± 0.015	Yes
F_I	Fluorescence intensity at I-step (30 ms)	0.325 ± 0.014	0.305 ± 0.012	Yes
F_M	Maximal fluorescence intensity	0.379 ± 0.017	0.358 ± 0.014	Yes
V_J	Variable fluorescence at J step	0.680 ± 0.061	0.645 ± 0.080	No
V_I	Variable fluorescence at I step	0.664 ± 0.090	0.621 ± 0.048	No
M_0	Approximated initial slope of fluorescence transients	3.093 ± 0.240	3.200 ± 0.223	No
**Quantum yields**				
φ_{Po}	Maximum quantum yield for primary photochemistry of PSII	0.423 ± 0.021	0.395 ± 0.028	Yes
ψ_o	The efficiency that a trapped exciton moves an electron into the electron transport chain beyond Q_A (at t = 0)	0.320 ± 0.061	0.355 ± 0.080	No
φ_{Eo}	Quantum yield for electron transport (at t = 0)	0.135 ± 0.025	0.140 ± 0.035	No
φ_{Do}	Quantum yield of energy dissipation (at t = 0)	0.577 ± 0.021	0.605 ± 0.028	Yes
**Specific fluxes or specific activities**				
ABS/RC	Absorption flux per reaction center (RC)	10.864 ± 1.309	12.795 ± 2.024	Yes
TR_0/RC	Trapped energy flux per RC (at t = 0)	4.584 ± 0.525	5.018 ± 0.641	No
ET_0/RC	Electron transport flux per RC (at t = 0)	1.491 ± 0.424	1.818 ± 0.584	No
DI_0/RC	Dissipated energy flux per RC (at t = 0)	6.280 ± 0.863	7.777 ± 1.486	Yes

Table S2. The fitted kinetic parameters of TA spectroscopy of *Synechocystis*.

Samples	τ_1 (ps)	τ_2 (ps)
Syn6803	3.00	68.1
Syn6803+C60-DMePyI (0.1 mg mL^{-1})	1.98	53.6
Syn6803+C60-DMePyI (0.5 mg mL^{-1})	1.05	44.6
Syn6803+DCBQ (5 mM)	0.22	4.54
